# Comprehensive Investigations into the Oil Extraction Process of Yellowish and Blackish Sesame Varieties, Parameters Optimization, and Absorbance Spectra Characteristics

**DOI:** 10.3390/foods14193450

**Published:** 2025-10-09

**Authors:** Abraham Kabutey, Sonia Habtamu Kibret, Su Su Soe, Mahmud Musayev

**Affiliations:** Department of Mechanical Engineering, Faculty of Engineering, Czech University of Life Sciences Prague, 16500 Prague, Czech Republic; xkibs001@studenti.czu.cz (S.H.K.); xsoes001@studenti.czu.cz (S.S.S.); xmusm025@studenti.czu.cz (M.M.)

**Keywords:** bulk sesame seeds, factorial design, independent factors, oil yield, extraction losses, seedcake sediments

## Abstract

The demand for sesame oil is increasing due to its nutritious and medicinal qualities and industrial applications such as biodiesel production. Mechanical oil extraction is commonly used although yield is lower. Roasting conditions could improve oil yield. The present study investigated heating conditions (temperature: 40, 50, and 60 °C and time: 15, 30, and 45 min) on oil extraction parameters of yellowish and blackish sesame varieties under a screw pressing operation based on a factorial design involving twenty-six experimental runs. The determined amounts of moisture content of yellowish and blackish sesame samples were 3.49 ± 0.19% w.b. and 6.69 ± 0.07% w.b. In that order, the oil contents of the samples were 38.73 ± 2.61% and 45.31 ± 6.51%. The overall optimal factor levels for explaining the calculated parameters (weight loss, seedcake, sediments in the oil, extraction loss, extracted crude oil, oil yield, and oil expression efficiency) were the heating temperature of 50 °C and time of 22.5 min for yellowish sesame, whereas those of blackish sesame were 60 °C and 15 min. The determined regression models with the significant terms predicted the crude oil, oil yield, and oil expression efficiency of yellowish sesame with the amounts of 25.496 g, 25.806%, and 66.631% in comparison with blackish sesame with the amounts of 20.449 g, 22.215%, and 49.029%. Yellowish sesame produced higher oil output than blackish sesame under the heating conditions. Similarities of absorption peaks were observed which can be used to assess adulteration and oil quality parameters.

## 1. Introduction

Edible oils from oilseeds are a vital component of the human diet, containing essential nutrients including fat, protein, fatty acids, chlorophyll, tocopherol, squalene, and carotenoids, which provide nutritional support for human health [1]. Common oilseeds include sunflower, pumpkin, flaxseed, and sesame [2,3]. Studies have demonstrated that incorporating oilseeds into various bakery products can enhance their antioxidant activity and can serve as functional foods satisfying the consumer demand for healthy and nutritious products [2,4,5,6,7].

Sesame (*Sesamum indicum* L.) belongs to the family Pedaliaceae which is considered as one of the oldest oilseeds and ranks second to olive oil [8]. Sesame seeds contain high oil content (44–60%), carbohydrates (18%), protein (18–25%), ash (5.7%), fiber (3.2%), essential minerals, a high amount of methionine, cysteine and tryptophan, secondary metabolites (lignans, saponins, flavonoids, and phenolic compounds), calcium, phosphorus, iron, and vitamins B and E [9,10,11,12,13,14,15,16]. Sesame is also rich in polyunsaturated fatty acids such as linoleic acid, oleic acid, palmitic acid, and stearic acid, which are primary fatty acids in sesame [8]. Sesame oil is considered the queen of oils due to its nutritional and medicinal qualities [10,13]. The oil has been utilized to produce some industrial products including ointment, paints, margarine, varnishes, and as a feedstock for biodiesel production [17,18,19,20]. According to [12], about 70% of the world’s sesame seeds are used to produce oil and meal, and the total annual oil and food consumption is about 65% and 35%, respectively. Presently, the demand for sesame seeds/oil is increasing primarily due to consumers’ consumption patterns and increasing health awareness [12,14]. The sesame market is expected to reach US $17.77 billion by the end of 2025 [14]. Sesame is mostly grown in Africa and Asia on a global scale, producing approximately 95.9% whereas America and Europe produce the remaining 4.1% [14]. In 2020, the top ten sesame-producing countries were Sudan, Myanmar, Tanzania, India, Nigeria, China, Mainland, Burkina Faso, Chad, Ethiopia, and South Sudan [14]. Presently, the top ten sesame seed producing countries in the world are India (production volume: over 1.5 million metric tons), China (around 1 million metric tons), Sudan (around 800,000 metric tons), Myanmar (around 600,000 metric tons), Tanzania (around 500,000 metric tons), Ethiopia (around 400,000 metric tons), Nigeria (around 300,000 metric tons), Uganda (around 250,000 metric tons), Mali (around 200,000 million metric tons), and Burkina Faso (around 150,000 metric tons) [21].

In the literature, many studies have been conducted on sesame, ref. [9] tested six BINA and two BARI-developed sesame varieties, plus one local variety, on saline conditions by identifying the best-performing varieties. Ref. [14] investigated sesame production constraints and variety traits preference in southeastern Tanzania to bridge a knowledge vacuum by disclosing sesame cultivation bottlenecks and desired variety characteristics amongst Tanzanian growers and other participants in the value chain to encourage variety uptake and improvement, ref. [10] also evaluated land suitability for sesame production in Diga district, Western Ethiopia. Contrary to the above-mentioned studies, ref. [22] elucidated mechanisms through the structural and functional analyses of sesame proteins and supports the optimization of extraction processes by hypothesizing that roasting-induced changes in sesame protein structure enhance oil yield by emulsification capacity; Ref. [23] examined the interactive effect of roasting temperature and roasting time on sesame seeds using response surface methodology to obtain the optimal roasting processing conditions. Ref. [24] evaluated the effect of conventional oven roasting treatment on the physicochemical quality attributes of sesame seeds obtained from different regions. In these latter studies, it is evident that roasting thus influences the oil-holding capacity of sesame proteins, thereby altering the oil content, fatty acid composition, and physicochemical properties of the extracted sesame oil. Particularly, ref. [25] cited in [22], mentioned that roasting primarily functions to denature proteins through heating, which facilitates the coalescence of microscopic oil droplets within the cells, enhancing oil extraction efficiency. Consequently, oil yield is directly related to the extent of protein denaturation, which is influenced by the roasting temperature and duration. Sesame roasting is carried out to promote more flavor, desired color, and texture changes that enhance overall palatability [26]. However, high-temperature roasting has a significant effect on the structure and functional properties of sesame oil [22,25,27,28]. Most of the studies on sesame oil extraction mentioned above focused on the yellowish-white variety under roasting temperatures above 60 °C, which has a negative effect on the quality of the oil. A systematic assessment of roasting conditions (temperature and time) at lower levels is necessary to understand their effect on oil yield, physicochemical properties, flavor quality, among other attributes of sesame seed oil. In addition, the comparison of yellowish-white sesame with different varieties under mechanical screw processing is essential to determine their oil yield efficiency and to determine the optimum roasting conditions to preserve its nutritional properties.

Therefore, the present study examined two varieties of sesame (yellowish and blackish), aimed at determining the mass of extracted oil, oil yield, and oil expression efficiency; the study identifies the optimal roasting conditions (heating temperature and time) based on a factorial design, and evaluates the seedcake sediments in the oil, oil extraction losses, and extraction throughput under mechanical screw pressing. The absorbance–wavelength spectra curves of the oil samples at various heating temperatures and heating times were evaluated.

## 2. Materials and Methods

### 2.1. Samples

**Figure 1:**
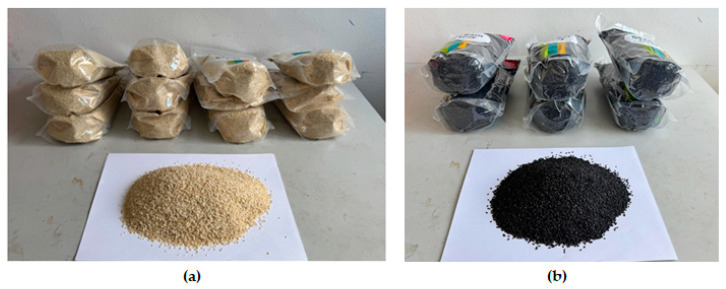
Samples of (**a**) yellowish sesame and (**b**) blackish sesame.

Samples of yellowish and blackish sesame (Figure 1a,b) were procured from Vitalcountry.cz., Plzenská, Štěnovice, Czech Republic. The samples were packaged in tight plastic bags and kept under laboratory conditions of a temperature of 24 ± 1 °C and a humidity of 44 ± 2%.

### 2.2. Moisture Content Determination

The moisture content of the samples was determined using the hot-air oven procedure by drying the samples at a temperature of 105 °C and a drying time of 17 h [29]. An electronic balance (KERN & SOHN 440–35, Balingen, Germany) with an accuracy of 0.01 g was used for the samples’ weight measurement. The moisture content of the samples was calculated using Equation (1) [30].

(1) $$MC=\left(\frac{m_{bf}-m_{af}}{m_{bf}}\right)\times 100$$

where MC is the moisture content in wet basis (%), $m_{bf}$ is the mass of the sample before drying, and $m_{af}$ is the mass of the sample after oven drying. The measurements were performed in triplicate and the results were averaged.

### 2.3. Oil Content Determination

The oil content of the samples was determined using the Soxhlet extraction procedure [31,32,33]. Following the procedure, the mass of each sample was measured at 10 g using an electronic weighing balance. The measured sample was ground using a mini grinder. The ground sample was packed into a thimble and placed in a Soxhlet extractor attached to a 500 mL round-bottom flask containing 500 mL of petroleum ether. The oil extraction process was allowed for 8 h with several extraction cycles. After the 8 h cycle duration, the extracted oil was dried in an oven for 5 h at 60 °C to remove the residual solvent in the extracted oil. The procedure was performed in duplicate, and the results were presented as the mean and standard deviation. The oil content of the samples was calculated using Equation (2) [31,32].

(2) $$OC=\left[\left(\frac{E_{OIL}}{M_{S}}\right)\times 100\right]$$

where OC is the oil content in (%), $E_{OIL}$ is the extracted seed oil after the Soxhlet extraction procedure, and $M_{S}$ is the initial mass of the sample. The measurements were performed twice and the results were averaged.

### 2.4. Factorial Design of Experiment

The factorial design of the input factors at three levels (heating temperature: 40, 50, and 60 °C, and heating time: 15, 30, and 45) generated nine experimental runs plus four replications of the center values (Table 1) for each variety of sesame (yellowish and blackish). In all, 26 experimental runs were performed.

The factor levels were coded from −1 (low value) to +1 (high value) with 0 as the center value according to Equation (3) [34,35].

(3) $$x_{i}=\frac{X_{i}-X_{0}}{∆X}$$

where $x_{i}$ is the coded value of the *i*th variable, $X_{i}$ is the uncoded value of the *i*th test variable, $X_{0}$ is the uncoded value of the *i*th test variable at the center point, and ∆X is the step change in the real value of the variable *i* corresponding to the variation in a unit for the dimensionless value of the variable *i*. The mathematical equation defining the factorial design is given in Equation (4) [36].

(4) $$Y=\beta_{0}+\sum_{i=1}^{k}\beta_{i}X_{i}+\sum_{i=1}^{k}\beta_{ii}X_{i}^{2}+\sum_{i_{1_{<j}}}^{k}\sum_{j}^{k}\beta_{ij}X_{i}X_{j}$$

where Y is the response variable; $\beta_{0},\;\beta_{i}$, $\beta_{ii}$, and $\beta_{ij}$ are the regression coefficients of the intercept, linear, quadratic, and interaction terms, respectively; $X_{i}$ and $X_{j}$ are the independent variables; and k is the number of factors.

### 2.5. Pretreatment of Samples and Oil Extraction Procedure

**Figure 2:**
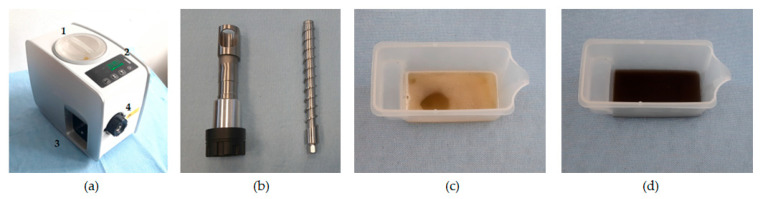
(**a**) Yoda electric oil press; 1: hopper; 2: panel for oilseed selection and pressing button; 3: crude oil recovery chamber; and 4: seedcake exit through the screw cage pin; (**b**) screw shaft and casing; (**c**) extracted yellowish sesame crude oil; and (**d**) extracted blackish sesame crude oil.

**Figure 3:**
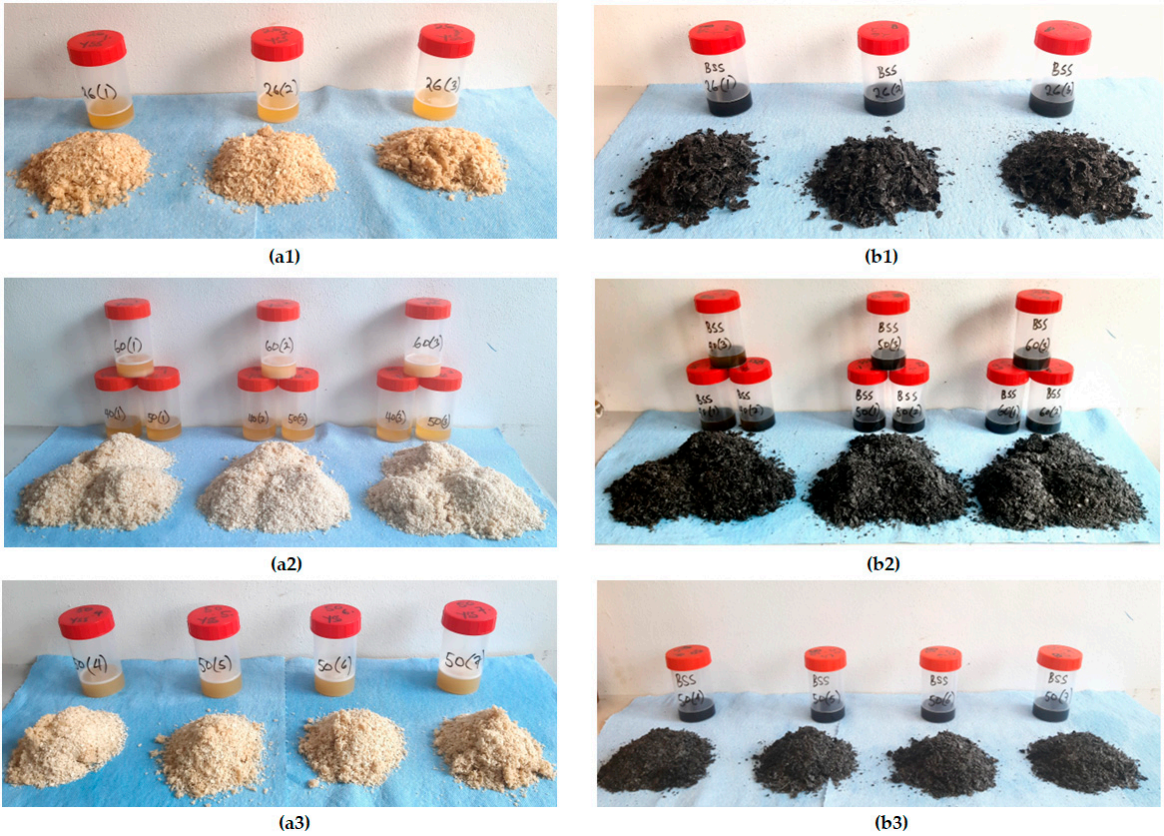
Extracted crude oil with sediments following the factorial design for yellowish sesame (**a1**–**a3**) and blackish sesame (**b1**–**b3)** for the control temperature of 24 °C (**a1**,**b1**) and heating temperatures of 40 °C, 50 °C, and 60 °C (**a2**,**a3**,**b2**,**b3**) for various heating times of 15, 30, and 45 min.

The samples of yellowish and blackish sesame were separately subjected to pretreatment or heating conditions following the factorial design (Table 1). For each experimental run, 100 g of the sample was used (100 g × 26 runs = 2600 g = 2.6 kg). The oil extraction process was performed using the Yoda electric oil press (Figure 2a). The screw press shaft and casing are shown in Figure 2b. The oil press is powered by an electric motor with a voltage rating of 220–240 V/50 HZ. The motor power is 180 W, and the heating power is 330 W. The preliminary oil extraction from yellowish and blackish sesame is shown in Figure 2c and Figure 2d, respectively. The experimental runs (Table 1) were not replicated since an optimum outcome can be achieved with the factorial design or any other optimization design of experiments. The extracted crude oils at the various heating conditions for the sesame varieties are shown in Figure 3.

### 2.6. Measurement of Absorbance–Wavelength Values

The absorbance versus wavelength curves in relation to the heating temperatures and heating times were obtained using an FTIR Alpha II spectrometer (Bruker Optics GmbH & Co. KG, Ettlingen, Germany) equipped with an attenuated total reflectance (ATR) accessory with a zinc-selenide crystal. The spectra of the oil samples were recorded by 16 scans at 4 cm^−1^, and the background spectrum without a sample was performed every 20 min to remove instrumental and atmospheric contributions to the spectrum of a sample [37,38]. For each analysis, only one drop of oil was required. The surface of the crystal was cleaned with acetone before the background and subsequent measurements [39]. The oil samples under control and heating conditions were analyzed in triplicate, and a mean absorbance–wavelength spectrum was obtained using Microsoft Excel.

### 2.7. Calculated Responses from the Oil Extraction Process

#### 2.7.1. Mass of Extracted Crude Oil

The mass of extracted crude oil was determined as the difference between the initial mass of the sample and the seed/press cake. The extracted crude oils contained seedcake sediments, which were also estimated.

#### 2.7.2. Mass of Extracted Oil and Seedcake Sediments

**Figure 4:**
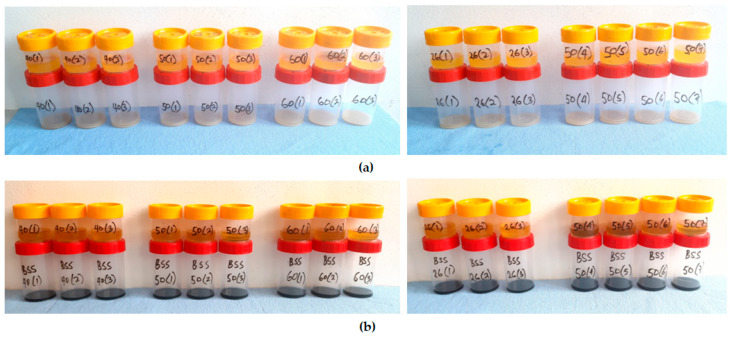
Extracted oil at the top and sediments at the bottom for (**a**) yellowish sesame and (**b**) blackish sesame for control temperatures of 24 °C and heating temperatures of 40 °C, 50 °C, and 60 °C for various heating times of 15, 30, and 45 min.

The extracted crude oils with the sediments were kept under laboratory conditions (temperature of 27 ± 1 °C and humidity of 40 ± 2%) for a week for the seedcake sediments to settle at the bottom of the oil container (Figure 4). Afterwards, a siphon tube was used to recover the oil atop, and the mass of oil and the seedcake sediments were measured using the electronic balance mentioned above.

#### 2.7.3. Percentage Oil Yield

The oil yield of the samples was calculated using Equation (5) [36,40].

(5) $$OY=\left[\left(\frac{M_{OL}}{M_{S}}\right)\times 100\right]$$

where OY is oil yield (%), $M_{OL}$ is the mass of oil determined as the difference between mass of the seedcake and the initial mass of the sample $M_{S}$ (g).

#### 2.7.4. Percentage Oil Expression Efficiency

The oil expression efficiency of the samples was calculated using Equation (6) [41].

(6) $$OEF=\left[\left(\frac{OY}{OC}\right)\times 100\right]$$

where OEF is the oil expression efficiency (%) and OC is the percentage of oil content (%) in the sample determined by the Soxhlet extraction procedure [32].

#### 2.7.5. Extraction Loss

The extraction loss (extracted crude oil and seedcake sediments) was calculated using Equation (7) [42].

(7) $$EL=\left[\frac{W_{ms}-{(W}_{Or}+W_{rc})}{W_{ms}}\times 100\right]$$

where EL is the extraction loss (%), $W_{ms}$ is the mass of the sample after pretreatment (g), $W_{Or}$ is the mass of extracted crude oil (g), and $W_{rc}$ is the mass of residual cake (g).

#### 2.7.6. Throughput

The throughput was calculated using Equation (8) [43].

(8) $$TP=\frac{W_{t}}{T_{m}}$$

where TP is the throughput in kg/h, $W_{t}$ is the sample weight in kg, and $T_{m}$ is the oil extraction time (s) recorded using a digital stopwatch.

### 2.8. Statistical Analysis

The experimental data were subjected to statistical analysis by employing general linear models and response surface regression techniques using STATISTICA 13 software [44]. The graphical illustrations were also performed by the above-mentioned software.

## 3. Results

### 3.1. Determined Moisture and Oil Contents

The determined amounts of the moisture content of yellowish and blackish sesame samples were 3.49 ± 0.19% w.b. and 6.69 ± 0.07% w.b., whereas the oil content amounts were 38.73 ± 2.61% and 45.31 ± 6.51%. It was observed that yellowish sesame had a lower moisture content and oil content than blackish sesame. The ratio of the oil yield to the oil content explains the oil expression efficiency. The effect of moisture content on the oil extraction process and oil expression efficiency is discussed in Section 4.

### 3.2. Evaluation of the Determined Parameters of Yellowish Sesame

The determined parameters of yellowish sesame are presented in Table 2 and Table 3. The control data, without heating conditions, are given in Table 2, whereas the heating conditions (heating temperature and time) data following the factorial design (Table 1) are given in Table 3. Here, the sample weight of 100 g at control conditions remained constant, meaning there was no pretreatment, hence no weight loss before the oil extraction process. However, for the heating conditions, there was a reduction in sample weight ranging from 97.71 g to 99.55 g. The parameters of the oil extraction process evaluated were the extraction time (s), throughput (g/s), seedcake output (g), extracted crude oil with seedcake sediments (g), total amount of seedcake and extracted crude oil with sediments (g), percentage extraction losses during (*) and after (**) extraction process (%), seedcake sediments in the oil (g), extracted crude oil without seedcake sediments (g), oil yield (%), oil expression efficiency (%), percentage extraction losses (***) during the separation of sediments and oil (%), and percentage total extraction losses (%). The data in Table 2 were compared with Table 3 to understand the effect of heating conditions of yellowish sesame on the determined parameters.

Based on the results of the main effects ANOVA analysis (see Supplementary Materials, Tables S1–S3), the heating conditions did not significantly (*p*-value > 0.05) affect the extraction time, throughput, seedcake sediments in the oil, and all the extraction losses both during and after the extraction process. However, the heating temperature significantly (*p*-value < 0.05) affected the seedcake output, extracted oil without the seedcake sediments, oil yield, and oil expression efficiency in comparison with the heating time, which did not significantly (*p*-value > 0.05) affect those parameters. Nevertheless, the heating conditions (heating temperature and time) significantly affected the extracted crude oil with sediment.

### 3.3. Evaluation of the Determined Parameters of Blackish Sesame

The determined parameters of blackish sesame are presented in Table 4 and Table 5. The control data without the heating pretreatment are given in Table 4, whereas the heating conditions data following the factorial design (Table 1) are given in Table 5. The data in Table 4 were compared with Table 5 to understand the heating conditions (heating temperature and time) of blackish sesame on the determined parameters mentioned in the preceding section. The sample weight reduction values after the heating conditions ranged from 96.46 g to 98.52 g. Based on the results of the main effects ANOVA analysis (see Supplementary Materials, Tables S4–S6), the extraction time, throughput, seedcake output, extracted crude oil, and the extraction loss after the extraction process were not affected significantly (*p*-value > 0.05) by the heating conditions (heating temperature and time). However, the seedcake sediments in the oil, extracted oil without the seedcake sediments, oil yield, oil expression efficiency, the extraction losses during the transfer of the crude oil into the plastic containers, and cumulative amounts of the crude oil without sediments and sediments only were significantly affected (*p*-value < 0.05) by the heating temperature in comparison with the heating time which did not significantly (*p*-value > 0.05) affect those parameters.

### 3.4. Comparison of Oil Output Parameters of Sesame Varieties

The oil output parameters of sesame varieties (yellowish and blackish) were the extracted crude oil with seedcake sediments, extracted crude oil without seedcake sediments, oil yield, and oil expression efficiency. The extracted crude oil with seedcake sediments for yellowish sesame in relation to the extraction losses (oil and seedcake) across the processing conditions (control and heating pretreatments) ranged from 28.94 to 34.92 g. The extracted crude without seedcake sediments and oil yield ranged from 21.82 to 27.41 g and 22.08 to 27.69%. The oil expression efficiency ranged from 57.02 to 71.49%. In comparison with blackish sesame, the extracted crude oil with seedcake sediments ranged from 25.85 to 32.06 g. The extracted crude oil without seedcake sediments and oil yield ranged from 19.75 to 27.03 g and 20.25 to 27.03%. The oil expression efficiency ranged from 44.70 to 59.66%. From the values stated above, it was observed that the yellowish sesame oil output parameters were higher than blackish sesame. However, the increase in the heating conditions increased the output parameters among the sesame varieties. Based on the results of the *t*-test analysis across the processing conditions, there were significant differences (*p*-value < 0.05) among the oil output parameters of the two sesame varieties, as shown in Table 6. A higher absolute t-value indicates a greater difference between sample groups, suggesting that the null hypothesis, which states that there is no significant difference, may be rejected in favor of the alternative hypothesis, which states that there is a significant difference among the sample groups and vice versa.

### 3.5. Comparison of Extraction Losses of Sesame Varieties

The percentage extraction losses (oil and seedcake sediments) of yellowish and blackish sesame after the extraction process, during the transfer of the crude oil into plastic containers, and during the separation of the crude oil without seedcake sediments and seedcake sediments were compared. All the extraction losses for yellowish sesame ranged from 0.05 to 2.42% whereas the blackish sesame ranged from 0.04 to 2.45%. These extraction losses for yellowish sesame were lower than blackish sesame across the processing conditions, showing a negative correlation or decreasing trends. Based on the *t*-test results across the processing conditions, there were no significant differences among the percentage extraction losses during the transfer and separation stages of the two sesame varieties, as shown in Table 7. However, the mean difference in the percentage extraction losses of the two sesame varieties after the extraction process was significant (*p*-value < 0.05). A higher absolute t-value indicates a greater difference between sample groups, suggesting that the null hypothesis, which states that there is no significant difference, may be rejected in favor of the alternative hypothesis, which states that there is a significant difference among the sample groups and vice versa.

### 3.6. Comparison of Seedcake, Sediments, and Throughput of Sesame Varieties

The amounts of seedcake, seedcake sediments in the oil, and the throughput of yellowish sesame across the processing conditions ranged from 64.20 to 69.41 g, 4.13 to 8.47 g, and 0.37 to 0.42 g/s, respectively. The blackish sesame amounts for seedcake ranged from 66.46 to 70.39 g, seedcake sediments ranged from 3.11 to 5.99 g, and throughput ranged from 0.41 to 0.44 g/s. These amounts increased with the heating conditions for both sesame varieties. However, seedcake and throughput amounts were higher for blackish sesame than for yellowish sesame. On the other hand, the amounts of seedcake sediments in the oil for yellowish sesame were higher than blackish sesame. Based on the *t*-test results across the processing conditions, there were significant differences among the dependent parameters, as shown in Table 8. A higher absolute t-value indicates a greater difference between sample groups, suggesting that the null hypothesis, which states that there is no significant difference, may be rejected in favor of the alternative hypothesis, which states that there is a significant difference among the sample groups and vice versa.

### 3.7. Comparison of Weight Losses of Sesame Varieties

As already mentioned in Section 3.2 and Section 3.3, the sesame samples showed a reduction in weight under the heating conditions. Based on the *t*-test results across the processing conditions, there were significant differences among the dependent parameters, as shown in Table 9. A higher absolute t-value indicates a greater difference between sample groups, suggesting that the null hypothesis, which states that there is no significant difference, may be rejected in favor of the alternative hypothesis, which states that there is a significant difference among the sample groups and vice versa.

### 3.8. Determined Regression Models of Dependent Parameters of Sesame Varieties

The response surface regression analysis focused on the main parameters from the several parameters described in Section 3.1, Section 3.2, Section 3.3, Section 3.4, Section 3.5, Section 3.6 and Section 3.7. These parameters included weight loss, seedcake, sediments in the oil, extraction loss, extracted crude oil, oil yield, and oil expression efficiency of yellowish and sesame varieties. The determined regression models are provided in Table 10. The corresponding standard error values, as well as the detailed analysis of variance of the parameters in determining the adequacy of the regression models, are provided in the Supplementary Materials, Tables S7–S12. The standard error of the coefficient measures the precision of the estimates of the model coefficient. The ratio of the model coefficient to the standard error obtains the t-value. Many authors appear to use sampling standard deviation and standard error interchangeably [45]. Both are measures of spread. The higher the number, the more the data spread out [46]. In other words, the smaller the standard error, the more precise the model or parameter estimates. The observed (experimental data from Table 3 and Table 5), predicted (using the parameter regression models’ coefficients in Table 10), and residuals (difference between observed and predicted) are presented in Supplementary Materials, Tables S13 and S14. Further interpretation of the results is discussed in Section 4.

### 3.9. Determined Optimal Input Factor Levels of the Parameters of Sesame Varieties

**Figure 5:**
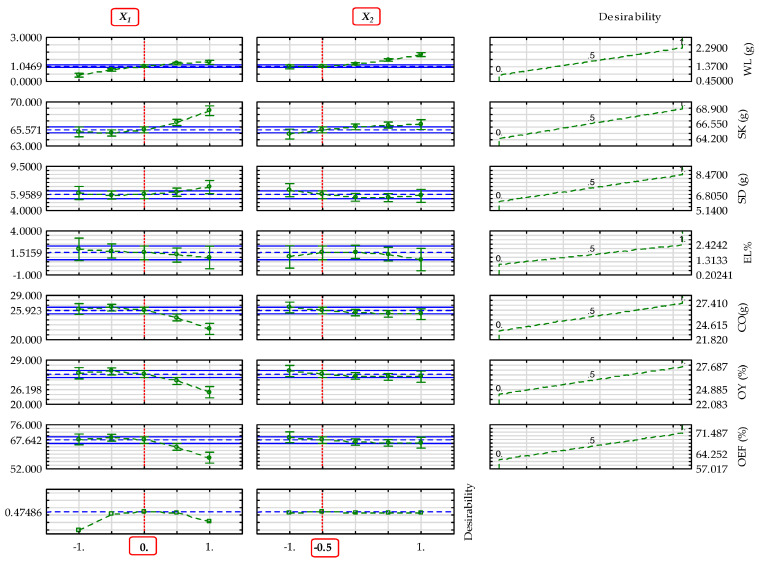
Profiles for predicted values and desirability of the factors’ effect on the dependent parameters of yellowish sesame—WL: weight loss; SK: seedcake; SD: sediments; EL: extraction loss; CO: extracted crude oil without seedcake sediments; OY: oil yield; and OEF: oil expression efficiency. The blue grid lines indicate the optimal values of the dependent parameters. The red gridlines indicate the optimal factor levels: $X_{1}$: heating temperature; $X_{2}$: heating time; coded value **0**: 50 °C and −**0.5**: 22.5 min.

**Figure 6:**
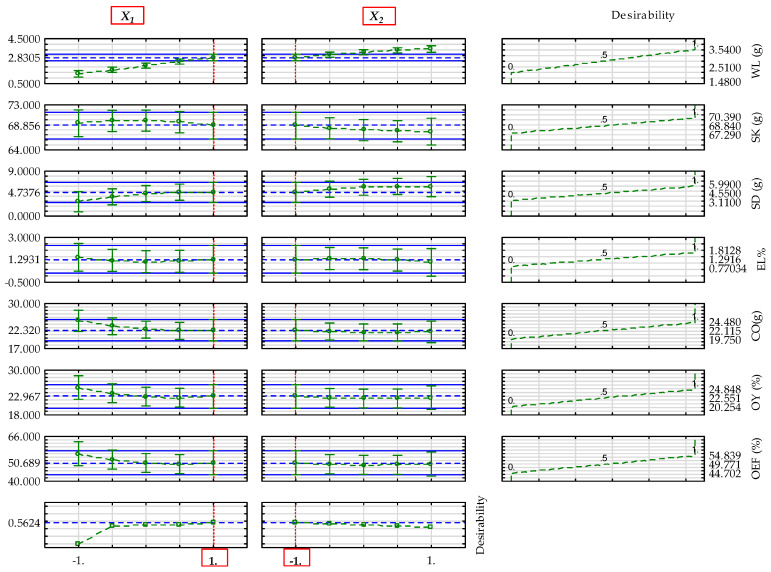
Profiles for predicted values and desirability of the factors’ effect on the dependent parameters of blackish sesame—WL: weight loss; SK: seedcake; SD: sediments; EL: extraction loss; CO: extracted crude oil without seedcake sediments; OY: oil yield; and OEF: oil expression efficiency. The blue grid lines indicate the optimal values of the dependent parameters. The red gridlines indicate the optimal factor levels: $X_{1}$: heating temperature; $X_{2}$: heating time; coded value +**1**: 60 °C and −**1**: 15 min.

The determined optimal input factor levels of the main parameters of yellowish and blackish sesame varieties mentioned above regarding the input factors are provided in Table 11 and Table 12. The values of desirability, profiles predicted, and models predicted are also provided. The profiles predicted that the values included both the significant and non-significant terms of the regression model, whereas the model predicted values considered only the significant terms of the regression model, as given in Table 10. The optimal factor levels were based on the individual desirability profiles of the dependent parameters of sesame varieties (Supplementary Materials, Figures S1–S14), whereas the overall optimal factor levels focused on the combined predicted and desirability profiles, as shown in Figure 5 and Figure 6. The desirability values range from 0 to 1, and higher value signify optimum outcome.

### 3.10. Absorbance Spectral Curves for Yellowish Sesame Oils

The results of the multivariate regression analysis of the absorbance versus wavelength data of yellowish sesame oils in relation to the heating conditions (heating temperatures: 40, 50, and 60 °C and heating times: 15, 30, and 45) are presented in Table 13.

**Figure 7:**
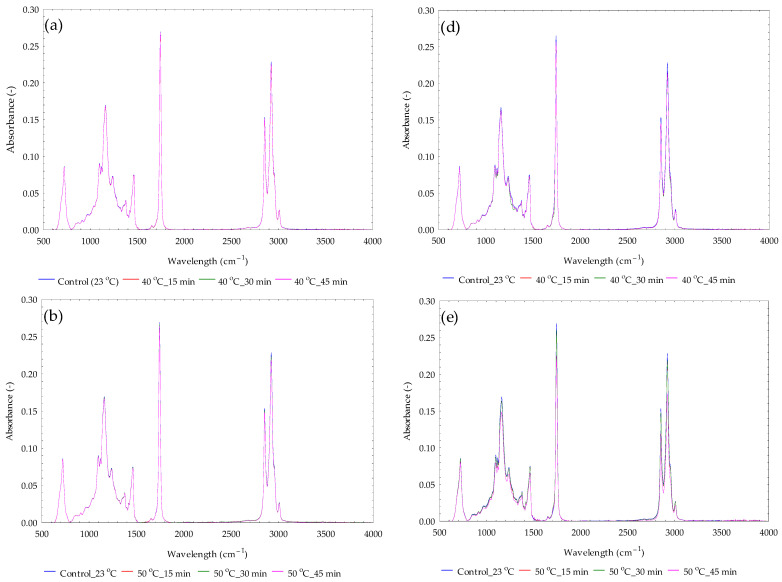

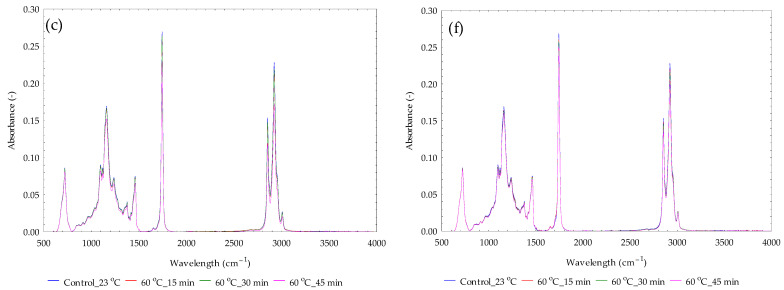
Absorbance versus wavelength of yellowish sesame oils (**a**–**c**) and blackish sesame oils (**d**–**f**) at control temperature and heating temperatures of 40 °C, 50 °C, and 60 °C for various heating intervals of 15, 30, and 45 min.

The absorbance–wavelength curves of yellowish sesame oils at various temperatures and heating times are illustrated in Figure 7a–c. It was noticed that the heating conditions did not significantly (*p*-value > 0.05) affect the absorbance values. However, the absorbance values showed both increasing and decreasing trends in relation to the independent factors. The absorbance values slightly increased with the heating temperature from 40 °C to 50 °C and substantially decreased from 50 °C to 60 °C. A similar observation was seen regarding the heating time. A negative correlation between the absorbance values and wavelength range was established. The correlation value was −0.283. The linear equation describing the relationship between the absorbance value (ABS) and wavelength (WL) of yellowish sesame oil (YSO) at the various heating temperatures and times is given in Equation (9).

(9) $${ABS}_{YSO}=0.0407-1.0303^{10-5}\times WL$$

### 3.11. Absorbance Spectral Curves for Blackish Sesame Oils

The results of the multivariate regression analysis of the relationship between absorbance and wavelength of blackish sesame oils in relation to the heating temperature and heating time are presented in Table 14.

The absorbance–wavelength curves of blackish sesame oils at various temperatures and heating times are illustrated in Figure 7d–f. It was observed that the heating temperature and heating time did not significantly (*p*-value > 0.05) affect the absorbance values. However, the absorbance values decreased from 40 °C to 50 °C and then increased from 50 °C to 60 °C. The absorbance values in relation to the heating time slightly decreased from 15 min to 30 min and considerably decreased from 30 min to 45 min. A negative correlation was found between the absorbance values and the wavelength range. The correlation value was −0.2853. The linear equation describing the relationship between the absorbance value (ABS) and wavelength (WL) of blackish sesame oil (YSO), dependent on the heating temperature and time, is given in Equation (10).

(10) $${ABS}_{YSO}=0.0404-1.0000^{10-5}\times WL$$

## 4. Discussion

In this study, two sesame varieties (yellowish and blackish) were investigated under control and heating conditions (heating temperature and heating time). In the preceding sections, detailed descriptions of the results were provided. In this section, however, emphasis is given to the main parameters, including the sample weight loss after heating conditions, seedcake after oil extraction, seedcake sediments in the extracted crude oil, percentage extraction loss, and the oil output parameters (extracted crude oil without the seedcake sediments, oil yield, and the oil expression efficiency) of the sesame varieties. In addition, the effect of the heating conditions on the main parameters, the response surface regression analysis for predicting the observed parameters, the determination of the optimal heating conditions for achieving the optimal oil output parameters, as well as the effect of the heating conditions on the absorbance spectrum are discussed.

Firstly, the increase in the heating conditions increased the amount of moisture in the seeds. Higher moisture reduction or weight loss was found in the blackish sesame seeds than in the yellowish sesame seeds. The mean weight loss for yellowish sesame was 1.08 ± 0.07 g, whereas the blackish sesame’s mean weight loss was 2.54 ± 0.16 g. Since the initial mass of the samples before the heating pretreatments was 100 g, the amounts of weight loss (g) were the same as the percentage moisture content on a weight basis. Moisture or water content is a measurement of the total water contained in a food product. To avoid microbial growth, the moisture content must be kept below 10% [47]. Moisture also impacts the stability of oils during storage. When higher moisture levels are present, hydrolysis reactions can take place that are accelerated or catalyzed by heat or residual enzymes, with the resultant free fatty acids being less stable to autooxidation than the triacylglycerols, leading to off-flavors, rancidity, and a reduced smoke point of the oils [48].

Secondly, the mean amounts of seedcake obtained from the yellowish and blackish sesame at the control conditions were 69.41 ± 0.17 g and 66.46 ± 0.30 g. With the heating conditions, the mean seedcake amounts were 66.49 ± 1.37 g and 68.98 ± 0.90 g. It can be stated that a higher amount of seedcake relates to a lower amount of extracted crude oil. Yellowish sesame in control conditions recorded a lower amount of the extracted crude oil, with the amount of 29.77 ± 0.23 g, compared to the blackish sesame, which produced a higher amount of 32.06 ± 0.15 g. However, under heating conditions, blackish sesame produced a higher amount of seedcake, hence, a lower amount of extracted crude oil. This means that heating pretreatment enhanced the oil output of yellowish sesame compared to blackish sesame. On the other hand, blackish sesame’s extracted crude oil was higher under control conditions without any pretreatment compared to yellowish sesame. Their mean amounts under heating conditions were 31.89 ± 1.37 g and 27.85 ± 0.81 g. Using Equations (5) and (6), the oil yield and oil expression efficiency of the sesame varieties were calculated. In the control conditions, yellowish sesame produced an oil yield of 24.80 ± 1.30% and oil expression efficiency of 64.04 ± 3.37%. The amounts of these parameters at the heating conditions were 25.18 ± 02% and 65.01 ± 4.36%. For blackish sesame at the control conditions, the amounts were 27.03 ± 0.75% and 59.66 ± 1.66% whereas at the heating conditions, the amounts were 22.97 ± 1.41% and 50.69 ± 3.11%. The oil expression efficiency encompasses the extracted crude oil, oil yield, and oil content in the sesame seeds. The oil expression efficiency for yellowish sesame was higher than blackish sesame at both control and heating conditions. The extracted crude oils of the sesame varieties contained seedcake sediments. The yellowish sesame produced an amount of 4.13 ± 1.10 g of seedcake sediments in the oil at the control conditions and 6.17 ± 0.85 g at the heating conditions. The corresponding amounts of the oil without the seedcake sediments for both processing conditions were 24.80 ± 1.30 g and 24.88 ± 1.74 g. On the other hand, the blackish sesame produced 4.12 ± 0.85 g seedcake sediments in the oil at the control conditions compared to 4.89 ± 1.11 g at the heating conditions. The corresponding amounts of oil without the seedcake sediments were 27.03 ± 0.75 g and 22.41 ± 1.47 g, respectively. It was observed that the heating conditions increased the seedcake sediments in the oil for both sesame varieties compared to the control conditions. The percentage extraction losses, which included the extracted crude oil and seedcake amounts, were higher at control conditions than heating conditions among the sesame varieties, indicating a positive effect of the heating conditions on oil production from the sesame varieties. The overall losses for processing the sesame varieties included the sample weight loss (the same as the percentage moisture loss on a weight basis) during the pretreatment process and the extraction loss during the oil extraction process. Cumulatively, these amounts ranged from 2.46 ± 0.61 to 3.73 ± 0.55 g, indicating that the amounts between 96.27 ± 0.55 g and 97.54 ± 0.61 g represented the extracted crude oil with seedcake sediments and seedcake, which together equal to the initial sample weight of 100 g. These parameters provide useful information on the efficiency of the Yoda electric oil press for processing edible oil from the sesame seeds.

Thirdly, in view of the literature’s perspective, edible oil production could be classified into cold-pressed (control conditions) and hot-pressed (heating conditions). In general, the hot-pressed method can achieve higher oil yield compared to the cold-pressed process [49,50,51]. However, hot-pressed oil production results in protein denaturation and solubility reduction [50,52]. On the other hand, cold-pressed processing can preserve natural beneficial components such as flavor, bioactive compounds, among others, in the oil due to the avoidance of the denaturation of the fats, proteins, carbohydrates, and lipids during the high-temperature pressing process [50,53]. These two oil production techniques are usually performed with mechanical pressing, which is a widely used oil extraction method favored for its operational simplicity, cost-effectiveness, and absence of organic solvents [49,54]. In this study, the mechanical oil press (Yoda electric oil press) produced a higher efficiency of the percentage oil output with lower percentage extraction loss in relation to the heating conditions. The overall optimal heating conditions for yellowish sesame were a temperature of 50 °C and a time of 22.5 min, producing an oil yield of 25.806% whereas blackish sesame optimal factor levels were a temperature of 60 °C and a time of 15 min, achieving an oil yield of 22.15%. In the literature, similar studies have been reported. Ref. [23] identified optimal roasting conditions for sesame at 180 °C for 10 min for optimal sesame oil production. The authors reported that the oil extraction rate increased with the increase in roasting temperature and time. High temperature tends to favor oil extraction rate since it decreases the moisture content of seeds and the viscosity of extracted oil [23,55,56]. Ref. [23] and ref. [57] also stated that sesame seeds absorbed high energy at high temperatures, which allowed for a stronger vibration of the polar substances in the cell, enhancing the oil extraction rate. Ref. [57] further indicated that by increasing the drying temperature from 70 to 105 °C, the oil yield of sesame varieties: off white, black, and brown increased by 2.5%, 5.5%, and 2.5%, respectively. The authors again mentioned that the off white was found to be the most suitable variety of sesame seed for maximum oil yield (49.5%) after 4 h of extraction using n-hexane.

Fourthly, based on the determined coefficients from the regression analysis of the observed parameters of sesame varieties (Table 10), the predicted and the residual values were estimated (Supplementary Materials, Tables S13 and S14). The lower the residual sum of squared values, the higher the suitability of the regression model is for predicting the observed data and vice versa. Basically, a random scatter of residuals around the zero line suggests that the regression model is appropriate and the normality assumption is achieved. The residuals’ normality assumption was further tested using the Shapiro–Wilk test, where the *p*-values were greater than the significance level of 0.05 with high coefficients of determination (R^2^) values between 0.880 and 0.969 (Supplementary Materials, Table S15). In general, the values of the coefficient of determination (R^2^) of the regression models ranged between 0.322 and 0.971. The corresponding correlation coefficients ranged from 0.567 to 0.985 (Table 11). Moreso, the adequacy of the regression models could be assessed based on the non-significance of the lack-of-fit *p*-values. The regression models established for yellowish sesame were satisfactory except for the ‘seedcake sediments in the oil’ regression model, which was unsatisfactory due to the significance of the lack-of-fit *p*-values (Supplementary Materials, Tables S10–S12). In contrast, all the regression models for blackish sesame were adequate (lack-of-fit *p* value > 0.05). The adequacy of the regression models also meant that only the significant coefficients of the intercept, linear, quadratic, and interaction of the input factors were used to determine the optimal factor levels and their responses (Table 11 and Table 12 and Figure 5 and Figure 6). It was observed that the heating conditions did not significantly affect the coefficients of the linear, quadratic, and interaction of the input factors of the blackish sesame parameters compared to the yellowish sesame regression models, which were significantly affected by the heating conditions. Ref. [55] reported that the correlation coefficient or the coefficient of determination of the regression model should be high, and the lack of fit *p*-value should not be significant.

Lastly, FTIR (Fourier Transform Infrared) spectroscopy has been used to assess vegetable oil adulteration [39,58,59,60,61]. The absorbance–wavelength spectra shown in Figure 7 for the yellowish and blackish sesame oils at control and heating conditions exhibited spectra similarities, indicating that the variations in heating temperature and heating time did not cause significant differences in the absorbance spectra. Usually, the characteristics of the absorption spectra for all vegetable oils include stretching vibrations of C(sp^3^)—H at 2854 cm^−1^ and 2922 cm^−1^, C=O at 1744 cm^−1^, C—O at 1150 cm^−1^, and 1108 cm^−1^, bending of CH_2_— at 1462 cm^−1^ and —CH_3_ at 1378 cm^−1^ [39]. Particularly, ref. [60] reported that at higher wavenumbers, stretching occurs with the double bonds associated with the structure of vegetable oils. In general, the absorption peaks described by Ref. [60] for the vegetable oils (hazelnut oil, canola oil, sunflower oil, and sesame oil) under cold-pressed conditions were similar to the peaks detected for the sesame oil varieties under the cold-pressed and heating conditions presently studied (Figure 7). Ref. [62] described more absorption peaks for the corn oil sample at a room temperature of 25 °C. Ref. [63] characterized physicochemical and thermo-oxidative properties of inaja fruit oil using FTIR and chromatographic techniques. These authors indicated that the peaks at 3008 cm^−1^, 2923 cm^−1^, and 2854 cm^−1^ are attributed to the stretching of hydrogen bonds. The peaks at 2962 cm^−1^ and 2872 cm^−1^ are linked to the symmetric and asymmetric stretching vibration shoulder of the aliphatic CH_3_ group. The peak at 1744 cm^−1^ represents the stretching of the ester carbonyl functional group (C=O) of the triglycerides. The absorption peaks in the wavelength range from 2000 to 1500 cm^−1^, which are associated with axial deformation vibrations of double bonds and the angular deformation of N–H and –NH_2_. The peaks around 1462 cm^−1^ and 1377 cm^−1^ result from bending vibrations of the CH_2_ and CH_3_ groups and the rocking vibrations of CH bonds of *cis*-disubstituted olefins. The peaks at 1377 cm^−1^ and 1160 cm^−1^ are responsible for bending CH_2_ groups. The peak at 1098 cm^−1^ is assigned to the stretching vibration of the C—O ester group. The peak at 722 cm^−1^ ascribes to rocking vibration of methylene (—CH_2_) and out-of-plane vibration of *cis*-disubstituted olefins. Similar descriptions were reported by ref. [64] on 103 FTIR spectra of eight types and 16 brands of edible oils datasets. It is important to mention that FTIR spectroscopy can be used to measure the oxidation state of edible oils and determine oil quality control parameters, including peroxide value and acidity index [61]. Again, ref. [61] reported that the bands at 3000 cm^−1^ contain a greater amount of unsaturated fatty acids, specifically oleic and linoleic acids. The authors also stated that the medium wavelength range of 1750 cm^−1^ is where the effects of the acidity of the oil and fat are observed with high absorbance.

## 5. Conclusions

The specific findings of the study, by evaluating the effect of heating conditions (temperature: 40, 50, and 60 °C and time: 15, 30, and 45 min) based on the factorial design on oil extraction parameters of yellowish and blackish sesame varieties were as follows. The percentage weight loss of the sesame samples during the heating conditions increased with an increase in the heating conditions. The percentage weight loss of the sesame samples implied a reduction in moisture content facilitating increased oil output. The seedcake after the oil production was higher in blackish sesame than in yellowish sesame, indicating a higher oil output in yellowish sesame than the blackish sesame. However, without the pretreatment process, that is, the control conditions, yellowish sesame obtained a higher seedcake than the yellowish sesame, leading to a higher oil output from blackish sesame than the yellowish sesame. The average amounts of the extracted crude oil under the heating conditions for both yellowish and blackish sesame varieties were 31.89 ± 1.61 g and 27.85 ± 0.81 g. Under the control conditions, the calculated amounts were 32.06 ± 0.15 g for blackish sesame and 29.77 ± 0.23 g for yellowish sesame. The seedcake sediments in blackish sesame increased along with the heating conditions, suggesting that the extracted crude oil without the seedcake sediments decreased along with the heating conditions. In comparison with yellowing sesame, the seedcake sediments in the oil and the extracted crude oil without the seedcake sediments showed both increasing and decreasing trends with the heating conditions. The percentage extraction loss of both sesame varieties reduced along with the heating conditions. The percentage extraction loss mean values at the control conditions for yellowish and blackish sesame were 1.69 ± 0.34% and 2.45 ± 0.15%. However, at the heating conditions, the mean value of the percentage extraction loss for yellowish sesame was 1.26 ± 0.63% and that of blackish sesame was 1.28 ± 0.32%. The optimal factor levels for obtaining the maximum oil expression efficiency of 74.13% from yellowish sesame were a temperature of 45 °C and heating time of 15 min with a desirability of 1.00. The blackish sesame factor levels were a temperature of 40 °C and heating time of 15 min with a desirability value of 0.953 achieving 49.03% of oil expression efficiency. The high desirability values indicate adequacy of the optimum factor levels. A quadratic model was suitable for describing the yellowish sesame parameters compared to the linear model which was suitable for the blackish sesame parameters. The established regression models’ lack-of-fit *p*-values were greater than the significance level of 0.05, indicating their adequacy for prediction. Finally, the absorbance–wavelength spectra for yellowish and blackish sesame oils at control and heating conditions showed similarities, indicating that the variations in heating temperature and heating time did not cause any significant differences. However, the absorbance peaks’ characteristics can be used to assess adulteration and oil quality control parameters such as peroxide value and acidity index. This information, together with the analysis of protein denaturation, oil release mechanisms, and the structural breakdown of sesame varieties will be explored in future studies.

## Figures and Tables

**Table 1:** Factorial design with 9 runs with 4 center points for processing sesame varieties.

Runs	Input $\mathrm{Factor},\;X_{1}$	Input $\mathrm{Factor},\;X_{2}$	Coded $\mathrm{Factor},\;X_{1}$	Coded $\mathrm{Factor},\;X_{2}$
1	40	15	−1	−1
2	40	30	−1	0
3	40	45	−1	1
4	50	15	0	−1
5	50	30	0	0
6	50	45	0	1
7	60	15	1	−1
8	60	30	1	0
9	60	45	1	1
10	50	30	0	0
11	50	30	0	0
12	50	30	0	0
13	50	30	0	0

$X_{1}$: heating temperature (°C) and $X_{2}$: heating time (min).

**Table 2:** Calculated parameters of yellowish sesame under control conditions.

$W_{t}$ (g)	$*Q_{1}$	$*Q_{2}$	$Q_{3}$	$Q_{4}$	$Q_{5}$	$Q_{6}$	$Q_{7}$
100	100	100	255.33 ± 7.23	0.39 ± 0.01	69.41 ± 0.17	29.77 ± 0.23 * 29.11 ± 0.24 ** 28.93 ± 0.21 ***	99.18 ± 0.39 * 98.52 ** ± 0.41 ** 98.34 ± 0.33 ***
$W_{t}$ **(g)**	$Q_{8}$	$Q_{9}$	$Q_{10}$	$Q_{11}$	$Q_{12}$	$Q_{13}$	* $Q_{14}$
100	4.13 ± 1.30	24.80 ± 1.30	24.80 ± 1.30	64.04 ± 3.37	(0.82 ± 0.39) ^A^ 0.82 ± 0.39 *	(1.48 ± 0.41) ^B^ 1.48 ± 0.41 **	(1.66 ± 0.33) ^C^ 1.69 ± 0.34 ***

$W_{t}$: initial sample weight (g), *$Q_{1}$: sample weight without pretreatment (g), *$Q_{2}$: sample without weight loss (g), $Q_{3}$: extraction time (s), $Q_{4}$: throughput (g/s), $Q_{5}$: seedcake output (g), $Q_{6}$: extracted crude oil with seedcake sediments, $Q_{7}$: total amounts of $Q_{5}$ and $Q_{6}$ (g), $Q_{8}$: seedcake sediments in the oil (g), $Q_{9}$: extracted crude oil without seedcake sediments (g), $Q_{10}$: oil yield (%), $Q_{11}$: oil expression efficiency (%), $Q_{12}$: percentage extraction loss (*) after the extraction process (Figure 2), $Q_{13}$: percentage extraction loss (**) during the transfer of the crude oil into plastic containers (Figure 2 and Figure 3), $Q_{14}$: percentage extraction loss (***) cumulative amounts of the crude oil without sediments and with sediments (g) (Figure 4), and ^A^, ^B^ and ^C^ represent the differences between $Q_{1}$ and $Q_{7}$ or the individual addition of ^A^, ^B^ and ^C^ to $Q_{7}$ to obtain $W_{t}$/$Q_{1}$/ $Q_{2}$.

**Table 3:** Calculated parameters of yellowish sesame following the factorial design.

$W_{t}$ (g)	Input $\mathrm{Factor}\;X_{1}$	Input $\mathrm{Factor}\;X_{2}$	Coded Factor $X_{1}$	$\mathrm{Coded}\;\mathrm{Factor}\;X_{2}$	$Y_{1}$	$Y_{2}$	$Y_{3}$	$Y_{4}$	$Y_{5}$	$Y_{6}$	$Y_{7}$
100	40	15	−1	−1	99.55	0.45	266	0.37	64.20	34.92 * 34.23 ** 33.90 ***	99.12 * 98.43 ** 98.10 ***
100	40	30	−1	0	99.28	0.72	251	0.40	66.13	32.38 * 31.73 ** 31.26 ***	98.51 * 97.86 ** 97.39 ***
100	40	45	−1	1	99.07	0.93	247	0.40	66.81	31.86 * 31.38 ** 31.52 ***	98.67 * 98.19 ** 98.33 ***
100	50	15	0	−1	99.00	1.00	243	0.41	64.86	33.41 * 32.77 ** 32.80 ***	98.27 * 97.63 ** 97.66 ***
100	50	30	0	0	98.81	1.19	243	0.41	65.73	32.39 * 31.80 ** 31.88 ***	98.12 * 97.53 ** 97.61 ***
100	50	45	0	1	97.71	2.29	237	0.41	66.22	31.30 * 31.07 ** 30.91 ***	97.13 * 97.29 ** 97.13 ***
100	60	15	1	−1	98.81	1.19	241	0.41	68.32	30.29 * 30.20 ** 30.29 ***	98.45 * 98.52 ** 98.61 ***
100	60	30	1	0	98.21	1.79	237	0.41	68.46	29.41 * 28.81 ** 28.43 ***	97.87 * 97.27 ** 96.89 ***
100	60	45	1	1	98.08	1.92	246	0.40	68.90	28.94 * 28.36 ** 28.41 ***	97.84 * 97.26 ** 97.31 ***
100	50	30	0	0	98.87	1.13	247	0.40	66.74	32.08 * 31.56 ** 31.30 ***	98.82 * 98.30 ** 98.04 ***
100	50	30	0	0	99.00	1.00	244	0.41	65.80	32.20 * 31.86 ** 30.80 ***	98.00 * 97.66 ** 96.60 ***
100	50	30	0	0	98.88	1.12	246	0.40	66.09	32.62 * 32.47 ** 30.72 ***	98.71 * 98.56 ** 96.81 ***
100	50	30	0	0	98.89	1.11	238	0.42	66.11	32.72 * 32.24 ** 31.40 ***	98.83 * 98.35 ** 97.51 ***
$W_{t}$ **(g)**	**Input** **Factor** $X_{1}$	**Input** **Factor** $X_{2}$	**Coded Factor** $X_{1}$	**Coded Factor** $X_{2}$	$Y_{8}$	$Y_{9}$	$Y_{10}$	$Y_{11}$	$Y_{12}$	$Y_{13}$	$Y_{14}$
100	40	15	−1	−1	6.95	26.95	27.07	69.90	(0.43) ^A^ 0.43 *	(1.12) ^B^ 1.13 **	(1.45) ^C^ 1.46 ***
100	40	30	−1	0	6.31	24.95	25.13	64.89	(0.77) ^A^ 0.78 *	(1.42) ^B^ 1.43 **	(1.89) ^C^ 1.90 ***
100	40	45	−1	1	5.87	25.65	25.89	66.85	(0.40) ^A^ 0.40 *	(0.88) ^B^ 0.89 **	(0.74) ^C^ 0.75 ***
100	50	15	0	−1	5.39	27.41	27.69	71.49	(0.73) ^A^ 0.74 *	(1.37) ^B^ 1.38 **	(1.34) ^C^ 1.35 ***
100	50	30	0	0	5.49	26.39	26.71	68.96	(0.69) ^A^ 0.70 *	(1.28) ^B^ 1.30 **	(1.20) ^C^ 1.21 ***
100	50	45	0	1	6.54	24.37	24.94	64.40	(0.19) ^A^ 0.19 *	(0.42) ^B^ 0.43 **	(0.58) ^C^ 0.59 ***
100	60	15	1	−1	8.47	21.82	22.08	57.02	(0.20) ^A^ 0.20 *	(0.29) ^B^ 0.29 **	(0.20) ^C^ 0.20 ***
100	60	30	1	0	5.74	22.69	23.10	59.65	(0.34) ^A^ 0.35 *	(0.94) ^B^ 0.96 **	(1.32) ^C^ 1.34 ***
100	60	45	1	1	6.28	22.13	22.56	58.26	(0.24) ^A^ 0.24 *	(0.82) ^B^ 0.84 **	(0.77) ^C^ 0.79 ***
100	50	30	0	0	5.97	25.33	25.62	66.15	(0.05) ^A^ 0.05 *	(0.57) ^B^ 0.58 **	(0.83) ^C^ 0.84 ***
100	50	30	0	0	5.90	24.90	25.15	64.94	(1.00) ^A^ 1.01 *	(1.34) ^B^ 1.35 **	(2.40) ^C^ 2.42 ***
100	50	30	0	0	5.14	25.58	25.87	66.80	(0.17) ^A^ 0.17 *	(0.32) ^B^ 0.32 **	(2.07) ^C^ 2.09 ***
100	50	30	0	0	6.18	25.22	25.50	65.85	(0.06) ^A^ 0.06 *	(0.54) ^B^ 0.55 **	(1.38) ^C^ 1.40 ***

$W_{t}$: initial sample weight (g), $X_{1}$: heating temperature (°C), $X_{2}$: heating time (min), $Y_{1}$: sample weight after pretreatment (g), $Y_{2}$: sample weight loss (g or %), $Y_{3}$: extraction time (s), $Y_{4}$: throughput (g/s), $Y_{5}$: seedcake (g), $Y_{6}$: extracted crude oil with seedcake sediments (*) after the extraction process (Figure 2), (**) during the transfer of crude oil into the plastic containers (Figure 2 and Figure 3), and (***) during the separation of crude oil without sediments and sediments (g) (Figure 4), and $Y_{7}$: total amounts of $Y_{5}$ and $Y_{6}$ (g). $Y_{8}$: seedcake sediments in the oil (g), $Y_{9}$: extracted crude oil without seedcake sediments (g), $Y_{10}$: oil yield (%), $Y_{11}$: oil expression efficiency (%), $Y_{12}$: percentage extraction loss (*) after the extraction process (Figure 2), $Y_{13}$: percentage extraction loss (**) during the transfer of the crude oil into the plastic containers (Figure 2 and Figure 3), $Y_{14}$: percentage extraction loss (***) during the separation of crude oil without sediments and sediments (g) (Figure 4), and ^A^, ^B^, and ^C^ represent the difference between $Y_{1}$ and $Y_{7}$ or the individual addition of ^A^, ^B^, and ^C^ to $Y_{7}$ to obtain $Y_{1}$.

**Table 4:** Calculated parameters of blackish sesame under control conditions.

$W_{t}$ (g)	$*Q_{1}$	$*Q_{2}$	$Q_{3}$	$Q_{4}$	$Q_{5}$	$Q_{6}$	$Q_{7}$
100	100	100	242.33 ± 11.02	0.41 ± 0.02	66.46 ± 0.30	32.06 ± 0.15 * 31.49 ± 0.14 ** 31.15 ± 0.23 ***	98.52 ± 0.20 * 97.95 ± 0.20 ** 97.61 ± 0.14 ***
$W_{t}$ **(g)**	$Q_{8}$	$Q_{9}$	$Q_{10}$	$Q_{11}$	$Q_{12}$	$Q_{13}$	* $Q_{14}$
100	4.12 ± 0.85	27.03 ± 0.75	27.03 ± 0.75	59.66 ± 1.66	(1.48 ± 0.20) ^A^ 1.48 ± 0.20 *	(2.05 ± 0.20) ^B^ 2.05 ± 0.20 **	(2.39 ± 0.14) ^C^ 2.45 ± 0.15 ***

$W_{t}$: initial sample weight (g), *$Q_{1}$: sample weight without pretreatment (g), *$Q_{2}$: sample without weight loss (g), $Q_{3}$: extraction time (s), $Q_{4}$: throughput (g/s), $Q_{5}$: seedcake output (g), $Q_{6}$: extracted crude oil with seedcake sediments, $Q_{7}$: total amounts of $Q_{5}$ and $Q_{6}$ (g), $Q_{8}$: seedcake sediments in the oil (g), $Q_{9}$: extracted crude oil without seedcake sediments (g), $Q_{10}$: oil yield (%), $Q_{11}$: oil expression efficiency (%), $Q_{12}$: percentage extraction loss (*) after the extraction process (Figure 2), $Q_{13}$: percentage extraction loss (**) during the transfer of the crude oil into plastic containers (Figure 2 and Figure 3), $Q_{14}$: percentage extraction loss (***) cumulative amounts of the crude oil without sediments and sediments only (Figure 4), and ^A^, ^B^, and ^C^ represent the differences between $Q_{1}$ and $Q_{7}$ or the individual addition of ^A^, ^B^, and ^C^ to $Q_{7}$ to obtain $W_{t}$/$Q_{1}$/ $Q_{2}$.

**Table 5:** Calculated parameters of blackish sesame following the factorial design.

$W_{t}$ (g)	Input $\mathrm{Factor}\;X_{1}$	Input $\mathrm{Factor}\;X_{2}$	$\mathrm{Coded}\;\mathrm{Factor}\;X_{1}$	$\mathrm{Coded}\;\mathrm{Factor}\;X_{2}$	$Y_{1}$	$Y_{2}$	$Y_{3}$	$Y_{4}$	$Y_{5}$	$Y_{6}$	$Y_{7}$
100	40	15	−1	−1	98.52	1.48	225	0.44	69.21	28.50 * 28.10 ** 27.81 ***	97.71 * 97.31 ** 97.02 ***
100	40	30	−1	0	98.34	1.66	222	0.44	69.35	28.14 * 27.57 ** 27.52 ***	97.49 * 96.92 ** 96.87 ***
100	40	45	−1	1	98.14	1.86	226	0.43	68.72	28.37 * 27.93 ** 27.81 ***	97.09 * 96.65 ** 96.53 ***
100	50	15	0	−1	98.08	1.92	224	0.44	69.51	27.96 * 27.55 ** 27.56 ***	97.47 * 97.06 ** 97.07 ***
100	50	30	0	0	97.4	2.6	226	0.43	69.37	27.48 * 27.01 ** 26.90 ***	96.85 * 96.38 ** 96.27 ***
100	50	45	0	1	97.16	2.84	223	0.44	69.27	27.38 * 26.98 ** 26.98 ***	96.65 * 96.25 ** 96.25 ***
100	60	15	1	−1	97.08	2.92	224	0.43	69.31	27.28 * 26.87 ** 26.51 ***	96.59 * 96.18 ** 95.82 ***
100	60	30	1	0	96.74	3.26	223	0.43	67.29	28.68 * 28.26 ** 28.18 ***	95.97 * 95.55 ** 95.47 ***
100	60	45	1	1	96.46	3.54	221	0.44	67.88	28.23 * 27.67 ** 27.55 ***	96.11 * 95.55 ** 95.43 ***
100	50	30	0	0	97.51	2.49	225	0.43	70.39	25.85 * 25.48 ** 25.52 ***	96.24 * 95.87 ** 95.91 ***
100	50	30	0	0	97.36	2.64	224	0.43	69.77	27.55 * 27.10 ** 26.84 ***	97.32 * 96.87 ** 96.61 ***
100	50	30	0	0	97.38	2.62	225	0.43	69.14	27.60 * 27.25 ** 27.26 ***	96.74 * 96.39 ** 96.40 ***
100	50	30	0	0	97.64	2.36	223	0.44	67.49	29.08 * 28.64 ** 28.38 ***	96.57 * 96.13 ** 95.87 ***
$W_{t}$ **(g)**	**Input** **Factor** $X_{1}$	**Input** **Factor** $X_{2}$	**Coded Factor** $X_{1}$	**Coded Factor** $X_{2}$	$Y_{8}$	$Y_{9}$	$Y_{10}$	$Y_{11}$	$Y_{12}$	$Y_{13}$	$Y_{14}$
100	40	15	−1	−1	3.33	24.48	24.85	54.84	(0.81) ^A^ 0.82 *	(1.21) ^B^ 1.23 **	(1.50) ^C^ 1.52 ***
100	40	30	−1	0	3.11	24.41	24.82	54.78	(0.85) ^A^ 0.86 *	(1.42) ^B^ 1.44 **	(1.47) ^C^ 1.49 ***
100	40	45	−1	1	3.64	24.17	24.63	54.35	(1.05) ^A^ 1.07 *	(1.49) ^B^ 1.52 **	(1.61) ^C^ 1.64 ***
100	50	15	0	−1	3.85	23.71	24.17	53.35	(0.61) ^A^ 0.62 *	(1.02) ^B^ 1.04 **	(1.01) ^C^ 1.03 ***
100	50	30	0	0	4.09	22.81	23.42	51.69	(0.55) ^A^ 0.56 *	(1.02) ^B^ 1.05 **	(1.13) ^C^ 1.16 ***
100	50	45	0	1	5.48	21.5	22.13	48.84	(0.51) ^A^ 0.52 *	(0.91) ^B^ 0.94 **	(0.91) ^C^ 0.94 ***
100	60	15	1	−1	4.94	21.57	22.22	49.04	(0.49) ^A^ 0.50 *	(0.90) ^B^ 0.93 **	(1.26) ^C^ 1.30 ***
100	60	30	1	0	5.73	22.45	23.21	51.22	(0.77) ^A^ 0.80 *	(1.19) ^B^ 1.23 **	(1.27) ^C^ 1.31 ***
100	60	45	1	1	5.75	21.80	22.60	49.88	(0.35) ^A^ 0.36 *	(0.91) ^B^ 0.94 **	(1.03) ^C^ 1.07 ***
100	50	30	0	0	5.77	19.75	20.25	44.70	(1.27) ^A^ 1.30 *	(1.64) ^B^ 1.68 **	(1.60) ^C^ 1.64 ***
100	50	30	0	0	5.99	20.85	21.42	47.26	(0.04) ^A^ 0.04 *	(0.49) ^B^ 0.50 **	(0.75) ^C^ 0.77 ***
100	50	30	0	0	5.98	21.28	21.85	48.23	(0.64) ^A^ 0.66 *	(0.99) ^B^ 1.02 **	(0.98) ^C^ 1.01 ***
100	50	30	0	0	5.89	22.49	23.03	50.84	(1.07) ^A^ 1.10 *	(1.51) ^B^ 1.55 **	(1.77) ^C^ 1.81 ***

$W_{t}$: initial sample weight (g), $X_{1}$: heating temperature (°C), $X_{1}$: heating time (min), $Y_{1}$: sample weight after pretreatment (g), $Y_{2}$: sample weight loss (g or %), $Y_{3}$: extraction time (s), $Y_{4}$: throughput (g/s), $Y_{5}$: seedcake (g), $Y_{6}$: extracted crude oil with seedcake sediments (*) after the extraction process (Figure 2), (**) during the transfer of crude oil into the plastic containers (Figure 2 and Figure 3) and (***) during the separation of crude oil without sediments and sediments only (g) (Figure 4), and $Y_{7}$: total amounts of $Y_{5}$ and $Y_{6}$ (g). $Y_{8}$: seedcake sediments in the oil (g), $Y_{9}$: extracted crude oil without seedcake sediments (g), $Y_{10}$: oil yield (%), $Y_{11}$: oil expression efficiency (%), $Y_{12}$: percentage extraction loss (*) after the extraction process (Figure 2), $Y_{13}$: percentage extraction loss (**) during the transfer of the crude oil into the plastic containers (Figure 2 and Figure 3), $Y_{14}$: percentage extraction loss (***) during the separation of crude oil without sediments and sediments only (g) (Figure 4), and ^A^, ^B^, and ^C^ represent the difference between $Y_{1}$ and $Y_{7}$ or the individual addition of ^A^, ^B^, and ^C^ to $Y_{7}$ to obtain $Y_{1}$.

**Table 6:** Results of *t*-test of oil output parameters of sesame varieties in relation to the processing conditions (control, heating temperature, and heating time).

Dependent Parameters	Mean	Std. Dev.	N	Mean Diff.	t-Value	df.	*p*-Value
YSS_CO_SK	31.735	1.646	14				
BSS_CO_SK	28.154	1.369	14	3.581	6.257	26	<0.05
YSS_CO_WSK	24.871	1.672	14				
BSS_CO_WSK	22.736	1.877	14	2.135	3.177	26	<0.05
YSS_OY	25.152	1.627	14				
BSS_OY	23.259	1.737	14	1.892	2.975	26	<0.05
YSS_OEF	64.942	4.201	14				
BSS_OEF	51.334	3.833	14	13.607	8.953	26	<0.05

YSS: yellowish sesame; BSS: blackish sesame; Std. Dev.: standard deviation; N: number of samples; Diff.: difference; df: degrees of freedom; CO_SK: extracted crude oil with seedcake sediments (g); CO_WSK: extracted crude oil without seedcake sediments (g); OY: oil yield (%); OEF: oil expression efficiency (%); df: degrees of freedom; and *p*-value < 0.05 implies significance.

**Table 7:** Results of *t*-test of extraction losses of sesame varieties in relation to the processing conditions (control, heating temperature, and heating time).

Dependent Parameters	Mean	Std. Dev.	N	Mean Diff.	t-Value	df.	*p*-Value
YSS_EL_1	0.439	0.313	14				
BSS_EL_1	0.765	0.382	14	−0.326	−2.468	26	<0.05
YSS_EL_2	0.923	0.431	14				
BSS_EL_2	1.222	0.393	14	−0.299	−1.919	26	>0.05
YSS_EL_3	1.289	0.616	14				
BSS_EL_3	1.367	0.436	14	−0.078	−0.388	26	>0.05

YSS: yellowish sesame; BSS: blackish sesame; Std. Dev.: standard deviation; N: number of samples; Diff.: difference; EL_1: percentage extraction losses after the extraction process (%); EL_2: percentage extraction losses during the transfer of the crude oil into plastic containers (%); and EL_3: percentage extraction losses during the separation of the crude oil without seedcake sediments and seedcake sediments (%); df: degrees of freedom; *p*-value < 0.05 implies significance and *p*-value > 0.05 implies non-significance.

**Table 8:** Results of *t*-test of seedcake, sediments in the oil, and throughput of sesame varieties in relation to the processing conditions (control, heating temperature, and heating time).

Dependent Parameters	Mean	Std. Dev.	N	Mean Diff.	t-Value	df.	*p*-Value
YSS_SK	66.698	1.533	14				
BSS_SK	68.797	1.098	14	−2.099	−4.164	26	<0.05
YSS_SD	6.026	0.979	14				
BSS_SD	4.833	1.089	14	1.192	3.047	26	<0.05
YSS_TP	0.403	0.011	14				
BSS_TP	0.434	0.007	14	−0.031	−9.302	26	<0.05

YSS: yellowish sesame; BSS: blackish sesame; Std. Dev.: standard deviation; N: number of samples; Diff.: difference; SK: seedcake (g); SD: seedcake sediments in the oil (g); TP: throughput (g/s); df: Degrees of freedom; and *p*-value < 0.05 implies significance.

**Table 9:** Results of *t*-test of seedcake weight loss of sesame varieties in relation to the processing conditions (control, heating temperature, and heating time).

Dependent Parameters	Mean	Std. Dev.	N	Mean Diff.	t-Value	df.	*p*-Value
YSS_SWL	98.879	0.581	14				
BSS_SWL	97.701	0.885	14	1.168	4.125	26	<0.05

YSS: yellowish sesame; BSS: blackish sesame; Std. Dev.: standard deviation; N: number of samples; Diff.: difference; SWL: sample weight loss (g); df: degrees of freedom; and *p*-value < 0.05 implies significance.

**Table 10:** Determined regression models for yellowish and blackish sesame varieties.

Effect	Yellowish Sesame: Parameter Regression Model Coefficients based on (Equation (4))						
	WL (g)	SK (g)	SD (g)	EL (%)	CO (g)	OY (%)	OEF (%)
Intercept	*1.202*	*66.050*	*5.648*	*1.622*	*25.496*	*25.806*	*66.631*
(L)	*0.467*	*1.423*	0.227	−0.296	*−* *1.818*	*−* *1.724*	*−* *4.451*
$X_{1}$ (Q)	*−* *0.177*	*1.354*	0.597	−0.069	*−* *1.707*	*−* *1.778*	*−* *4.591*
$X_{2}$ (L)	*0.417*	*0.758*	−0.353	−0.148	*−* *0.672*	−0.574	−1.483
$X_{2}$ (Q)	*0.213*	−0.401	0.537	−0.719	0.363	0.419	1.081
$X_{1}$ (L) by $X_{2}$ (Q)	0.063	−0.508	−0.278	0.323	0.403	0.415	1.072
**R^2^**	0.835	0.959	0.494	0.605	0.867	0.859	0.859
**R**	0.914	0.979	0.703	0.778	0.931	0.927	0.927
**Effect**	**Blackish Sesame: Parameter Regression Model Coefficients based on (Equation (4))**						
	WL **(g)**	SK **(g)**	SD **(g)**	EL **(%)**	CO **(g)**	OY **(%)**	OEF **(%)**
Intercept	*2.521*	*69.190*	*5.423*	*1.240*	*21.656*	*22.215*	*49.029*
$X_{1}$ (L)	*0.787*	−0.467	*1.057*	−0.163	−1.207	−1.045	−2.307
$X_{1}$ (Q)	−0.009	−0.766	−0.702	0.258	1.224	1.248	55
$X_{2}$ (L)	*0.320*	−0.360	0.458	−0.034	−0.382	−0.314	−0.693
$X_{2}$ (Q)	−0.089	0.304	−0.457	−0.163	0.399	0.385	0.851
$X_{1}$ (L) by $X_{2}$ (Q)	0.060	−0.235	0.125	−0.087	0.135	0.150	0.332
**R^2^**	0.971	0.400	0.746	0.322	0.627	0.584	0.584
**R**	0.985	0.633	0.864	0.567	0.792	0.764	0.764

$X_{1}$: heating temperature (°C), $X_{2}$: heating time (min); L: linear term; Q: quadratic term; R^2^: coefficient of determination; WL: weight loss; SK: seedcake; SD: sediments; EL: extraction loss; CO: extracted crude oil without seedcake sediments; OY: oil yield; OEF: oil expression efficiency, and the italicized values represent significant terms.

**Table 11:** Predicted values and desirability of yellowish sesame from the dependent parameters’ profiles.

Dependent Parameters (Blackish Sesame)	Optimal Factor Levels		Desirability Value	Profiles Predicted Value *	Model Predicted Value **
	$X_{1}$ (°C)	$X_{2}$ (min)			
WL (g)	+1 (60)	+1 (45)	0.942	2.183	2.085
SK (g)	+1 (60)	+0.5 (37.5)	0.989	68.853	68.953
SD (g)	+1 (60)	−1 (15)	0.751	7.639	5.648
EL (%)	−1 (40)	−0.5 (22.5)	0.766	1.904	1.622
CO (g)	−0.5 (45)	−1 (15)	0.965	27.215	27.259
OY (%)	−0.5 (45)	−1 (15)	0.953	27.424	27.458
OEF (%)	−0.5 (45)	−1 (15)	0.953	70.809	74.125
WL (g)	0 (50)	−0.5 (22.5)	0.475	1.047	0.994
SK (g)				65.571	65.671
SD (g)				5.959	5.648
EL (%)				1.516	1.622
CO (g)				25.923	25.496
OY (%)				26.198	25.806
OEF (%)				67.642	66.631

$X_{1}$: heating temperature (°C), $X_{2}$: heating time (min); WL: weight loss; SK: seedcake; SD: sediments; EL: extraction loss; CO: extracted crude oil without seedcake sediments; OY: oil yield; OEF: oil expression efficiency; * based on both the significant and non-significant terms of the model (Equation (4) and Table 10); and ** (based on only the significant terms of the model (Equation (4) and Table 10).

**Table 12:** Predicted values and desirability of blackish sesame from the *dependent parameters’* profiles.

Dependent Parameters (Blackish Sesame)	Optimal Factor Levels		Desirability Value	Profiles Predicted Value *	Model Predicted Value **
	$X_{1}$ (°C)	$X_{2}$ (min)			
WL (g)	+1 (60)	+1 (45)	1.000	3.591	3.628
SK (g)	0 (50)	−1 (15)	0.827	69.854	69.190
SD (g)	+1 (60)	+0.5 (37.5)	0.988	5.955	6.480
EL (%)	−1 (40)	0 (30)	0.855	1.661	1.240
CO (g)	−1 (40)	−1 (15)	1.000	25.003	22.863
OY (%)	−1 (40)	−1 (15)	1.000	25.359	22.215
OEF (%)	−1 (40)	−1 (15)	1.000	55.967	49.029
WL (g)	+1 (60)	−1 (15)	0.562	2.831	2.988
SK (g)				68.856	69.190
SD (g)				4.738	6.480
EL (%)				1.293	1.240
CO (g)				22.320	20.449
OY (%)				22.967	22.215
OEF (%)				50.689	49.029

$X_{1}$: heating temperature (°C), $X_{2}$: heating time (min); WL: weight loss; SK: seedcake; SD: sediments; EL: extraction loss; CO: extracted crude oil without seedcake sediments; OY: oil yield; OEF: oil expression efficiency; * based on both the significant and non-significant terms of the model (Equation (4) and Table 10); and ** (based on only the significant terms of the model (Equation (4) and Table 10).

**Table 13:** Multivariate results for the absorbance values of yellowish sesame oils.

Effect	Degree of Freedom	Sum of Squares	Mean Squares	*F*-Value	*p*-Value
Intercept	1	0.56573	0.565732	507.742	0.000000 *
Wavelength	1	1.44365	1.443649	1295.669	0.000000 *
Temperature	1	0.00229	0.002286	2.052	0.152048 **
Time	1	0.00113	0.001126	1.010	0.314829 **
Residual	14,891	16.59171	0.001114		
Total	14,894	18.03878			

* Significant (*p*-value < 0.05) and ** non-significant (*p*-value > 0.05).

**Table 14:** Multivariate results for the absorbance values of blackish sesame oil.

Effect	Degree of Freedom	Sum of Squares	Mean Squares	*F*-Value	*p*-Value
Intercept	1	0.48081	0.480807	456.499	0.000000 *
Wavelength	1	1.39028	1.390277	1319.991	0.000000 *
Temperature	1	0.00005	0.000050	0.048	0.152048 **
Time	1	0.00179	0.001788	1.697	0.314829 **
Residual	14,891	15.68391	0.001053		
Total	14,894	17.07603			

* Significant (*p*-value < 0.05) and ** non-significant (*p*-value > 0.05).

## Data Availability

The data presented in this study are available upon request from the corresponding author.

## Supplementary Materials

The following supporting information can be downloaded at: https://www.mdpi.com/article/10.3390/foods14193450/s1, Table S1: Univariate results of the determined parameters (extraction time and throughput) of yellowish sesame under the heating conditions; Table S2: Univariate results of the determined parameters (seedcake, extracted crude oil with seedcake sediments, sediments in the oil, extracted crude oil without seedcake sediments, and oil yield) of yellowish sesame under the heating conditions; Table S3: Univariate results of the determined parameters (oil expression efficiency and percentage extraction losses) of yellowish sesame under the heating conditions; Table S4: Univariate results of the determined parameters (extraction time, throughput, seedcake, extracted crude oil with seedcake sediments, and seedcake sediments in the oil) of blackish sesame under the heating conditions; Table S5: Univariate results of the determined parameters (extracted crude oil without seedcake sediments, oil yield, oil expression efficiency, and percentage extraction losses) of blackish sesame under the heating conditions; Table S6: Univariate results of the determined parameters (percentage extraction losses) of blackish sesame under the heating conditions; Table S7: Model parameter estimates (weight loss and seedcake) and their statistical evaluation for sesame varieties; Table S8: Model parameter estimates (seedcake sediments in the oil, extraction loss and extracted crude oil) and their statistical evaluation for sesame varieties; Table S9: Model parameter estimates (oil yield and oil expression efficiency) and their statistical evaluation for sesame varieties; Table S10. Analysis of variance for the weight loss and seedcake parameters (regression models) of sesame varieties; Table S11: Analysis of variance for the seedcake sediments in the oil and percentage extraction loss parameters (regression models) of sesame varieties; Table S12: Analysis of variance for the extracted crude oil, oil yield, and oil expression efficiency parameters (regression models) of sesame varieties; Table S13: Observed, predicted, and residual results of the determined parameters of yellowish sesame based on the regression coefficients (Equation (4) (Table 10)); Table S14: Observed, predicted, and residual results of the determined parameters of blackish sesame varieties based on the regression coefficients (Equation (4) (Table 10)); and Table S15: Shapiro–Wilk test of normality of residuals of the parameters of yellowish and blackish sesame varieties based on the regression coefficients (Equation (4) (Table 10)). Figure S1: Profiles for predicted values and desirability of the factors’ effect on weight loss (WL) of yellowish sesame ($X_{1}$: heating temperature; $X_{2}$: heating time; the blue grid lines indicate the optimal and desirability values of the dependent parameter, and the red gridlines indicate the factor levels; coded values +1: 60 °C and +1: 60 min); Figure S2: Profiles for predicted values and desirability of the factors’ effect on the seedcake (SK) of yellowish sesame ($X_{1}$: heating temperature; $X_{2}$: heating time; the blue grid lines indicate the optimal and desirability values of the dependent parameter, and the red gridlines indicate the factor levels; coded values +1: 60 °C and +0.5: 37.5 min); Figure S3: Profiles for predicted values and desirability of the factors effect on seedcake sediments (SD) of yellowish sesame ($X_{1}$: heating temperature; $X_{2}$: heating time; the blue grid lines indicate the optimal and desirability values of the dependent parameter, and the red gridlines indicate the factor levels; coded values +1: 60 °C and −1: 15 min); Figure S4: Profiles for predicted values and desirability of the factors effect on the extraction loss (EL) of yellowish sesame ($X_{1}$: heating temperature; $X_{2}$: heating time; the blue grid lines indicate the optimal and desirability values of the dependent parameter, and the red gridlines indicate the factor levels; coded values −1: 40 °C and −0.5: 22.5 min); Figure S5: Profiles for predicted values and desirability of the factors effect on the extracted crude oil (CO) of yellowish sesame ($X_{1}$: heating temperature; $X_{2}$: heating time; the blue grid lines indicate the optimal and desirability values of the dependent parameter, and the red gridlines indicate the factor levels; coded values −0.5: 45 °C and −1: 15 min). Figure S6: Profiles for predicted values and desirability of the factors’ effect on the oil yield (OY) of yellowish sesame ($X_{1}$: heating temperature; $X_{2}$: heating time; the blue grid lines indicate the optimal and desirability values of the dependent parameter, and the red gridlines indicate the factor levels; coded values −0.5: 45 °C and −1: 15 min); Figure S7: Profiles for predicted values and desirability of the factors’ effect on the oil expression efficiency (OEF) of yellowish sesame ($X_{1}$: heating temperature; $X_{2}$: heating time; the blue grid lines indicate the optimal and desirability values of the dependent parameter, and the red gridlines indicate the factor levels; coded values −0.5: 45 °C and −1: 15 min). Figure S8: Profiles for predicted values and desirability of the factors’ effect on the weight loss (WL) of blackish sesame ($X_{1}$: heating temperature; $X_{2}$: heating time; the blue grid lines indicate the optimal and desirability values of the dependent parameter, and the red gridlines indicate the factor levels; coded values +1: 60 °C and +1: 45 min); Figure S9: Profiles for predicted values and desirability of the factors’ effect on the seedcake (SK) of blackish sesame ($X_{1}$: heating temperature; $X_{2}$: heating time; the blue grid lines indicate the optimal and desirability values of the dependent parameter, and the red gridlines indicate the factor levels; coded values 0: 50 °C and −1: 15 min); Figure S10: Profiles for predicted values and desirability of the factors’ effect on the seedcake sediments (SD) of blackish sesame ($X_{1}$: heating temperature; $X_{2}$: heating time; the blue grid lines indicate the optimal and desirability values of the dependent parameter and the red gridlines indicate the factor levels; coded values +1: 60 °C and 0.5: 37.5 min); Figure S11: Profiles for predicted values and desirability of the factors’ effect on the extraction loss (EL) of blackish sesame ($X_{1}$: heating temperature; $X_{2}$: heating time; the blue grid lines indicate the optimal and desirability values of the dependent parameter, and the red gridlines indicate the factor levels; coded values −1: 40 °C and 0: 30 min); Figure S12: Profiles for predicted values and desirability of the factors’ effect on the extracted crude oil (CO) of blackish sesame ($X_{1}$: heating temperature; $X_{2}$: heating time; the blue grid lines indicate the optimal and desirability values of the dependent parameter, and the red gridlines indicate the factor levels; coded values −1: 40 °C and −1: 15 min); Figure S13: Profiles for predicted values and desirability of the factors’ effect on the oil yield (OY) of blackish sesame ($X_{1}$: heating temperature; $X_{2}$: heating time; the blue grid lines indicate the optimal and desirability values of the dependent parameter, and the red gridlines indicate the factor levels; coded values −1: 40 °C and −1: 15 min); and Figure S14: Profiles for predicted values and desirability of the factors effect on the oil expression efficiency (OEF) of blackish sesame ($X_{1}$: heating temperature; $X_{2}$: heating time; the blue grid lines indicate the optimal and desirability values of the dependent parameter, and the red gridlines indicate the factor levels; coded values −1: 40 °C and −1: 15 min).

## References

[1] Li X., Liu W., Xiao L., Zhao J., Chen Y., Zhang L., Li P., Perez-Marin D., Wang X. (2025). The application of emerging technologies for the quality and safety evaluation of oilseeds and edible oils. Food Chem. X.

[2] Slawinska N., Olas B. (2025). Effect of thermal processing on the antioxidant activity of oilseeds used in bakery products: A systematic review. Ind. Crops Prod..

[3] De Lamo B., Gomez M. (2018). Bread enrichment with oilseeds. A review. Foods.

[4] Esrafil M., Akter S., Alam M.J., Alim M.A., Reza M.S.A., Zubair M.A., Joy P.R., Jahan M., Khatun M. (2024). Development and quality evaluation of novel biscuits by utilizing fruits and vegetables seed. Food Res..

[5] Agirbas H.E.T., Yavuz-Duzgun M., Ozcelik B. (2022). Valorization of fruit seed flours: Rheological characteristics of composite dough and cake quality. J. Food Meas. Charact..

[6] Jiang X., Wang X., Zhou S. (2022). Effect of flaxseed marc flour on high-yield wheat bread production: Comparison in baking, staling, antioxidant and digestion properties. LWT—Food Sci. Technol..

[7] Tanska M., Rotkiewicz D. (2011). Quality of fat from oilseeds used to produce selected kinds of bread. Zywn. Nauka Technol. Jakosc Food Sci. Technol..

[8] Abdiani N., Kolahi M., Javaheriyan M., Sabaeian M. (2024). Effect of storage conditions on nutritional value, oil content, and oil composition of sesame seeds. J. Agric. Food Res..

[9] Khan M.S.U., Rahman M.M., Basak A.R., Angon P.B., Ritu S.A., Kobir M., Islam M.R. (2025). Evaluation of different sesame varieties cultivated under saline conditions in the southwestern coastal region of Bangladesh. Crop Des..

[10] Moisa M.B., Merga B.B., Gabissa B.T., Gemeda D.O. (2022). Assessment of land suitability for oilseeds crops (sesame and groundnut) using geospatial techniques: In the case of Diga district, East Wollega zone, western Ethiopia. Oil Crop Sci..

[11] Akondo M.R.I., Uddin F.J., Islam M.M., Adhikary S., Rana M.S. (2022). Yield comparison of bina developed four sesame varieties under the agro-ecological conditions of gopalganj district of Bangladesh. Res. Agric. Livest. Fish..

[12] Myint D., Gilani S.A., Kawase M., Watanabe K.N. (2020). Sustainable sesame (*Sesamum indicum* L.) production through improved Technology: An overview of production, challenges and opportunities in Myanmar. Sustainability.

[13] Bedigian D., Harlan J.R. (1986). Evidence for cultivation of sesame in the ancient world. Econ. Bot..

[14] Lukurugu G.A., Nzunda J., Kidunda B.R., Chilala R., Ngamba Z.S., Minja A., Kapinga F.A. (2023). Sesame production constraints, variety traits preference in the Southeastern Tanzania: Implication for genetic improvement. J. Agric. Food Res..

[15] Khazaei J., Mohammadi N. (2009). Effect of temperature on hydration kinetics of sesame seeds (*Sesamum indicum* L.). J. Food Eng..

[16] Obiajunwa E.I., Adebiyi F.M., Omode P.E. (2005). Determination of essential minerals and trace elements in Nigerian sesame seeds using TXRF technique. Pak. J. Nutr..

[17] Ashtiani S.-H.M., Emadi B., Sanaei-Moghadam A., Aghkhani M.-H. (2014). Effect of moisture content and temperature on thermal behaviour of sesame seed. AUDJG—Food Technol..

[18] Saydut A., Duz M.Z., Kaya C., Kafadar A.B., Hamamci C. (2008). Transestified sesame (*Sesame indicum* L.) seed oil as a biodiesel fuel. Bioresour. Technol..

[19] Orruno E., Morgan M.R.A. (2007). Purificatoin and characterization of the 7S globulin storage protein from sesame (*Sesame indicum* L.). Food Chem..

[20] Tunde-Akintunde T.Y., Akintunde B.O. (2004). Some physical properties of sesame seed. Biosyst. Eng..

[21] Top 10 Sesame Seed Producing Countries in the World in 2025. https://essfeed.com/top-10-sesame-seed-producing-countries-in-the/.

[22] Song G., Wang J., He Y., Sun Y., Huang J., Sun Q., Deng Z. (2025). Roasting increases the oil yield of sesame seeds by altering the amino acid composition and secondary structure of the sesame protein. Food Biosci..

[23] He S., Pan T., Zhang Z., Wu Y., Sun H., Ma Y., Zhang Y. (2023). Interactive effect of hot air roasting processes on the sensory property, allergenicity, and oil extraction of sesame (*Sesamum indicum* L.) seeds. Grain Oil Sci. Technol..

[24] Ahmed I.A.M., Uslu N., Ozcan M.M., Juhaimi F.A., Ghafoor K., Babiker E.E., Osman M.A., Alqah H.A.S. (2021). Effect of conventional oven roasting treatment on the physicochemical quality attributes of sesame seeds obtained from different regions. Food Chem..

[25] Elkhaleefa A., Shigidi I. (2015). Optimization of sesame oil extraction process conditions. ACES Adv. Chem. Eng. Sci..

[26] Kahyaoglu T., Kaya S. (2006). Modeling of moisture, color and texture changes in sesame seeds during the conventional roasting. J. Food Eng..

[27] Ma X., Li H., Zhang J., Ge Y., He L., Kang W., Huang W., Sun J.-L., Chen Y. (2022). Effect of roasting on the conformational structure and IgE binding of sesame allergens. J. Agric. Food Chem..

[28] Piravi-Vanak Z., Dadazadeh A., Azadmard-Damirchi S., Torbati M., Martinez F. (2024). The effect of extraction by pressing at different temperatures on sesame oil quality characteristics. Foods.

[29] (1996). Indian Standard Methods for Analysis of Oilseeds.

[30] Blahovec J. (2008). Agromaterials Study Guide.

[31] Danlami J.M., Arsad A., Zaini M.A.A. (2015). Characterization and process optimization of castor oil (*Ricinus communis* L.) extracted by the Soxhlet method using polar and non-polar solvents. J. Taiwan Inst. Chem Eng..

[32] Niu L., Li J., Chen M.-S., Xu Z.-F. (2014). Determination of oil contents in Sacha inchi (*Plukenetia volubilis*) seeds at different developmental stages by two methods: Soxhlet extraction and time-domain nuclear magnetic resonance. Ind. Crops Prod..

[33] Gürdil G.A.K., Kabutey A., Selvi K.Ç., Mizera Č., Herák D., Fraňková A. (2020). Evaluation of Postharvest Processing of Hazelnut Kernel Oil Extraction Using Uniaxial Pressure and Organic Solvent. Processes.

[34] Ocholi O., Menkiti M., Auta M., Ezemagu I. (2018). Optimization of the operating parameters for the extractive synthesis of biolubricant from sesame seed oil via response surface methodology. Egypt. J. Pet..

[35] Demirel C., Kabutey A., Herák D., Sedlaček A., Mizera Č., Dajbych O. (2022). Using Box–Behnken Design Coupled with Response Surface Methodology for Optimizing Rapeseed Oil Expression Parameters under Heating and Freezing Conditions. Processes.

[36] Chanioti S., Tzia C. (2017). Optimization of ultrasound-assisted extraction of oil from olive pomace using response surface technology: Oil recovery, unsaponifiable matter, total phenol content and antioxidant activity. LWT—Food Sci. Technol..

[37] Yuan L., Meng X., Xin K., Ju Y., Zhang Y., Yin C., Hu L. (2023). A Comparative Study on Classification of Edible Vegetable Oils by Infrared, near Infrared and Fluorescence Spectroscopy Combined with Chemometrics. Spectrochim. Acta Part A Mol. Biomol. Spectrosc..

[38] Mohammadi M., Khorrami M.K., Vatani A., Ghasemzadeh H., Vatanparast H., Bahramian A., Fallah A. (2020). Rapid Determination and Classification of Crude Oils by ATR-FTIR Spectroscopy and Chemometric Methods. Spectrochim. Acta Part A Mol. Biomol. Spectrosc..

[39] Nascimento T.A.d., Lopes T.I.B., Nazario C.E.D., Oliveira S.L., Alcantara G.B. (2021). Vegetable oils: Are they true? A point of view from ATR-FTIR, ^1^H NMR, and regiospecific analysis by ^13^ C NMR. Food Res. Int..

[40] Deli S., Farah Masturah M., Tajul Aris Y., Wan Nadiah W.A. (2011). The effects of physical parameters of the screw press oil expeller on oil yield from *Nigella sativa* L. seeds. Int. Food Res. J..

[41] Hernandez-Santos B., Rodriguez-Miranda J., Herman-Lara E., Torruco-Uco J.G., Carmona-Garcia R., Juarez-Barrientos J.M., Chavez-Zamudio R., Martinez-Sanchez C.E. (2016). Effect of oil extraction assisted by ultrasound on the physicochemical properties and fatty acid profile of pumpkin seed oil (*Cucurbita pepo*). Ultrason. Sonochem..

[42] Alenyorege E.A., Hussein Y.A., Adongo T.A. (2015). Extraction yield, efficiency and loss of the traditional hot water floatation (HWF) method of oil extraction from the seeds of allanblackia floribunda. Int. J. Sci. Technol. Res..

[43] Karaj S., Muller J. (2011). Optimizing mechanical oil extraction of *Jatropha curcas* L. seeds with respect to press capacity, oil recovery and energy efficiency. Ind. Crops Prod..

[44] Statsoft Inc. (2013). STATISTICA for Windows.

[45] Wooldridge J.M. (2023). What is standard error? (And how should we compute it?). J. Econom..

[46] Powell J.L. (2023). Discussion of “What is a standard error?”. J. Econom..

[47] Zambrano M.V., Dutta B., Mercer D.G., MacLean H.L., Touchie M.F. (2019). Assessment of moisture content measurement methods of dried food products in small-scale operations in developing countries: A review. Trends Food Sci. Technol..

[48] Meng X., Sedman J., van de Voort F.R. (2012). Improving the determination of moisture in edible oils by FTIR spectroscopy using acetonitrile extraction. Food Chem..

[49] Zhang C., Zhang L., Zhao Y., Cui R., Wu H., Xu M., Liu W., Liu R., Xu L., Song L. (2025). Effect of different heating pretreatment methods on physicochemical properties of pressed walnut oils and functional properties of walnut protein isolates. LWT—Food Sci. Technol..

[50] Dun Q., Yao L., Deng Z., Li H., Li J., Fan Y., Zhang B. (2019). Effects of hot and cold-pressed processes on volatile compounds of peanut oil and corresponding analysis of characteristic flavor components. LWT—Food Sci. Technol..

[51] Liu X., Jin Q., Liu Y., Huang J., Wang X., Mao W., Wang S. (2011). Changes in volatile compounds of peanut oil during the roasting process for production of aromatic roasted peanut oil. J. Food Sci..

[52] Zheng L., Ren J., Su G., Yang B., Zhao M. (2013). Comparison of in vitro digestion characteristics and antioxidant activity of hot-and cold-pressed peanut meals. Food Chem..

[53] Parker T.D., Adams D.A., Zhou K., Harris M., Yu L. (2010). Fatty acid composition and oxidative stability of cold-pressed edible seed oils. J. Food Sci..

[54] Ahmed I.A.M., Al-Juhaimi F.Y., Ozcan M.M., Osman M.A., Gassem M.A., Salih H.A.A. (2019). Effects of cold-press and Soxhlet extraction systems on antioxidant activity, total phenol contents, fatty acids, and tocopherol contents of walnut kernel oils. J. Oleo Sci..

[55] Bagheri H., Kashaninejad M., Ziaiifar A.M., Aalami M. (2019). Textural, color and sensory attributes of peanut kernels as affected by infrared roasting method. Inf. Process. Agric..

[56] Willems P., Kuipers N., De Haan A. (2008). Hydraulic pressing of oilseeds: Experimental determination and modelling of yield and pressing rates. J. Food Eng..

[57] Chakraborty D., Das J., Das P.K., Bhattacharjee S.C., Das S. (2017). Evaluation of the parameters affecting the extraction of sesame oil from sesame (*Sesamum indicum* L.) seed using soxhlet apparatus. Int. Food Res. J..

[58] Amit R.J., Kumari S., Dhaulaniya A.S., Balan B., Singh D.K. (2020). Application of ATR-FTIR spectroscopy along with regression modelling for the detection of adulteration of virgin coconut oil with paraffin oil. LWT—Food Sci. Technol..

[59] Uncu O., Ozen B. (2019). A Comparative study of mid-infrared, UV-Visible and fluorescence spectroscopy in combination with chemometric for the detection of adulteration of fresh olive oils with old olive oils. Food Control..

[60] Ozulku G., Yildirim R.M., Toker O.S., Karasu S., Durak M.Z. (2017). Rapid detection of adulteration of cold pressed sesame oil adulterated with hazelnut, canola, and sunflower oils using ATR-FTIR spectroscopy combined with chemometric. Food Control..

[61] Rizki H., Terouzi W., Kzaiber F., Hanine H., Oussama A. (2016). Quantification of adulterations in sesame oil with inferior edible oils by using ATR-FTIR coupled to chemometrics. J. Environ. Sci. Toxicol. Food Technol..

[62] Vlachos N., Skopelitis Y., Psaroudaki M., Konstantinidou V., Chatzilazarou A., Tegou E. (2006). Applications of Fourier transform-infrared spectroscopy to edible oils. Anal. Chim. Acta.

[63] Sim S.F., Ting W. (2012). An automated approach for analysis of Fourier Transform Infrared (FTIR) spectra of edible oils. Talanta.

[64] Rolim C.S.d.S., Freire J.O., Muniz I.d.C.B., Nunomura R.d.C.S., Santos L.S., Bauer L.C., Lamarao C.V., Bonomo R.C.F. (2024). Characterization of physiochemical and thermos oxidative properties of inaja fruit oil (*Maximiliana maripa*). Food Sci..

