# Supplementary material for: Comprehensive Investigations into the Oil Extraction Process of Yellowish and Blackish Sesame Varieties, Parameters Optimization, and Absorbance Spectra Characteristics
Source: Foods. 2025 Oct 9;14(19):3450. doi: 10.3390/foods14193450 (PMC12523327; doi:10.3390/foods14193450)
Supplement: Supplementary file 1 [file foods-14-03450-s001.zip › foods-3888410-supplementary.pdf]

foods

MDPI

Article (Supplementary Material)

Comprehensive Investigations into the Oil Extraction Process of Yellowish and Blackish Sesame Varieties, Parameters Optimization and Absorbance Spectra Characteristics

Abraham Kabutey*, Sonia Habtamu Kibret, Su Su Soe, Mahmud Musayev

Department of Mechanical Engineering, Faculty of Engineering, Czech University of Life Sciences Prague, 16500 Prague, Czech Republic; xkibs001@studenti.czu.cz (S.H.K.); xsoes001@studenti.czu.cz (S.S.S.); xmusm025@studenti.czu.cz (M.M.)

* Correspondence: kabutey@tf.czu.cz; Tel.: +420-22438-3180

Table S1. Univariate results of the determined parameters (extraction time and throughput) of yellowish sesame under the heating conditions.

Effect	Extraction time (s)				
	Degrees of freedom	Sum of squares	Mean squares	F-value	P-value
X_1	2	364.6667	182.3333	4.036900	0.109757
X_2	2	84.6667	42.3333	0.937269	0.463631
Error	4	180.6667	45.1667		
Total	9	708.4000			
**Effect**	**Throughput (g/s)**				
	**Degrees of freedom**	**Sum of squares**	**Mean squares**	**F-value**	**P-value**
X_1	2	0.000645	0.000323	2.974437	0.161649
X_2	2	0.000117	0.000059	0.541072	0.619478
Error	4	0.000434	0.000108		
Total	9	0.001332			

X_1: Heating temperature (°C), X_2: Heating time (min), P-value > 0.05 or lower F-value implies a non-significant effect.

Table S2. Univariate results of the determined parameters (seedcake, extracted crude oil with seedcake sediments, seedcake sediments in the oil, extracted crude oil without seedcake sediments, and oil yield) of yellowish sesame under the heating conditions.

Effect	Seedcake (g)				
	Degrees of freedom	Sum of squares	Mean squares	F-value	P-value
X_1	2	16.85749	8.428744	26.97534	0.004764
X_2	2	3.54869	1.774344	5.67861	0.067841
Error	4	1.24984	0.312461		
Total	9	28.63384			
	**Extracted crude oil with seedcake sediments (g)**				

Effect	Degrees of freedom	Sum of squares	Mean squares	F-value	P-value
X_1	2	20.72062	10.36031	36.56007	0.002690
X_2	2	7.39449	3.69724	13.04705	0.017667
Error	4	1.13351	0.28338		
Total	9	32.44841			

Effect	Seedcake sediments in the oil (g)				
	Degrees of freedom	Sum of squares	Mean squares	F-value	P-value
X_1	2	1.57762	0.788811	0.842002	0.495235
X_2	2	1.83442	0.917211	0.979061	0.450714
Error	4	3.74731	0.936828		
Total	9	11.54621			

Effect	Extracted crude oil without seedcake sediments (g)				
	Degrees of freedom	Sum of squares	Mean squares	F-value	P-value
X_1	2	28.03927	14.01963	12.39177	0.019312
X_2	2	2.71087	1.35543	1.19805	0.391102
Error	4	4.52547	1.13137		
Total	9	35.28344			

Effect	Oil yield (%)				
	Degrees of freedom	Sum of squares	Mean squares	F-value	P-value
X_1	2	26.97597	13.48799	12.48564	0.019063
X_2	2	1.98641	0.99321	0.91940	0.469325
Error	4	4.32112	1.08028		
Total	9	33.32571			

X_1: Heating temperature (°C), X_2: Heating time (min), P-value < 0.05 or higher F-value implies significant effect, and P-value > 0.05 or lower F-value implies non-significant effect.

Table S3. Univariate results of the determined parameters (oil expression efficiency and percentage extraction losses) of yellowish sesame under the heating conditions.

Effect	Oil expression efficiency (g)				
	Degrees of freedom	Sum of squares	Mean squares	F-value	P-value
X_1	2	179.8383	89.91913	12.48564	0.019063
X_2	2	13.2426	6.62131	0.91940	0.469325
Error	4	28.8072	7.20180		
Total	9	222.1695			

Effect	Percentage extraction loss after the extraction process (%)				
	Degrees of freedom	Sum of squares	Mean squares	F-value	P-value
X_1	2	0.152193	0.076096	2.525723	0.195292

X_2	2	0.159508	0.079754	2.647124	0.185222
Error	4	0.120514	0.030129		
Total	9	0.556558			

Effect	Percentage extraction loss during the transfer of the crude oil into the plastic containers (%)				
	Degrees of freedom	Sum of squares	Mean squares	F-value	P-value
X_1	2	0.333172	0.166586	1.188340	0.393487
X_2	2	0.392478	0.196239	1.399870	0.346047
Error	4	0.560735	0.140184		
Total	9	1.529807			

Effect	Percentage extraction loss during the separation of crude oil without sediments and sediments (%)				
	Degrees of freedom	Sum of squares	Mean squares	F-value	P-value
X_1	2	0.526241	0.263121	1.437441	0.338524
X_2	2	0.927552	0.463776	2.533631	0.194611
Error	4	0.732192	0.183048		
Total	9	2.535634			

Effect	Total percentage extraction losses (%)				
	Degrees of freedom	Sum of squares	Mean squares	F-value	P-value
X_1	2	2.71448	1.357241	1.709504	0.290689
X_2	2	3.93664	1.968318	2.479183	0.199371
Error	4	3.17575	0.793938		
Total	9	11.89274			

X_1: Heating temperature (°C), X_2: Heating time (min), P-value < 0.05 or higher F-value implies significant effect, and P-values > 0.05 or lower F-value implies non-significant effect.

Table S4. Univariate results of the determined parameters (extraction time, throughput, seedcake, extracted crude oil with seedcake sediments, and seedcake sediments in the oil) of blackish sesame under the heating conditions.

Effect	Extraction time (s)				
	Degrees of freedom	Sum of squares	Mean squares	F-value	P-value
X_1	2	5.5556	2.777778	0.675676	0.558718
X_2	2	1.5556	0.777778	0.189189	0.834629
Error	4	16.4444	4.111111		
Total	9	322.4000			

Effect	Throughput (g/s)				
	Degrees of freedom	Sum of squares	Mean squares	F-value	P-value
X_1	2	0.000027	0.000013	0.797550	0.511098
X_2	2	0.000001	0.000001	0.036048	0.964904

Effect	Degrees of freedom	Sum of squares	Mean squares	F-value	P-value
Error	4	0.000068	0.000017		
Total	9	0.000700			

Effect	Seedcake (g)				
	Degrees of freedom	Sum of squares	Mean squares	F-value	P-value
X_1	2	2.45176	1.225878	3.424771	0.135924
X_2	2	0.97396	0.486978	1.360484	0.354206
Error	4	1.43178	0.357944		
Total	9	10.12341			

Effect	Extracted crude oil with seedcake sediments (g)				
	Degrees of freedom	Sum of squares	Mean squares	F-value	P-value
X_1	2	0.81616	0.408078	1.329484	0.360833
X_2	2	0.05262	0.026311	0.085719	0.919493
Error	4	1.22778	0.306944		
Total	9	16.91556			

Effect	Seedcake sediments in the oil (g)				
	Degrees of freedom	Sum of squares	Mean squares	F-value	P-value
X_1	2	6.705689	3.352844	17.05896	0.011012
X_2	2	1.331356	0.665678	3.38691	0.137842
Error	4	0.786178	0.196544		
Total	9	8.912840			

X_1: Heating temperature (°C), X_2: Heating time (min), P-value > 0.05 or lower F-value implies a non-significant effect.

Table S5. Univariate results of the determined parameters (extracted crude oil without seedcake sediments, oil yield, oil expression efficiency, and percentage extraction losses) of blackish sesame under the heating conditions.

Effect	Extracted crude oil without seedcake sediments (g)				
	Degrees of freedom	Sum of squares	Mean squares	F-value	P-value
X_1	2	9.18436	4.592178	10.10281	0.027308
X_2	2	1.12136	0.560678	1.23349	0.382574
Error	4	1.81818	0.454544		
Total	9	26.82141			

Effect	Oil yield (%)				
	Degrees of freedom	Sum of squares	Mean squares	F-value	P-value
X_1	2	7.01829	3.509144	7.884161	0.040943
X_2	2	0.88494	0.442471	0.994121	0.446192
Error	4	1.78035	0.445088		
Total	9	20.51712			

**Effect**	**Oil expression efficiency (g)**				
	**Degrees of freedom**	**Sum of squares**	**Mean squares**	**F-value**	**P-value**
X_1	2	34.18559	17.09279	7.884161	0.040943
X_2	2	4.31049	2.15525	0.994121	0.446192
Error	4	8.67197	2.16799		
Total	9	99.99664			

**Effect**	**Percentage extraction loss after the extraction process (%)**				
	**Degrees of freedom**	**Sum of squares**	**Mean squares**	**F-value**	**P-value**
X_1	2	0.254277	0.127138	4.203723	0.103933
X_2	2	0.016421	0.008210	0.271472	0.775256
Error	4	0.120977	0.030244		
Total	9	0.965839			

**Effect**	**Percentage extraction loss during the transfer of the crude oil into the plastic containers (%)**				
	**Degrees of freedom**	**Sum of squares**	**Mean squares**	**F-value**	**P-value**
X_1	2	0.283837	0.141919	8.837465	0.034057
X_2	2	0.046928	0.023464	1.461137	0.333905
Error	4	0.064235	0.016059		
Total	9	1.130359			

X_1: Heating temperature (°C), X_2: Heating time (min), P-value > 0.05 or lower F-value implies a non-significant effect.

Table S6. Univariate results of the determined parameters (percentage extraction losses) of blackish sesame under the heating conditions.

**Effect**	**Percentage extraction loss during the separation of crude oil without sediments and sediments (%)**				
	**Degrees of freedom**	**Sum of squares**	**Mean squares**	**F-value**	**P-value**
X_1	2	0.400975	0.200487	14.03900	0.015549
X_2	2	0.017801	0.008900	0.62325	0.581276
Error	4	0.057123	0.014281		
Total	9	1.721308			

**Effect**	**Total percentage extraction losses (%)**				
	**Degrees of freedom**	**Sum of squares**	**Mean squares**	**F-value**	**P-value**
X_1	2	2.70495	1.352473	9.195294	0.031915
X_2	2	0.18668	0.093338	0.634591	0.576280
Error	4	0.58833	0.147083		
Total	9	10.93966			

X_1: Heating temperature (°C), X_2: Heating time (min), P-value < 0.05 or higher F-value implies significant effect, and P-values > 0.05 or lower F-value implies non-significant effect.

Table S7. Model parameter estimates (weight loss and seedcake) and their statistical evaluation for sesame varieties.

Effect	Yellowish Sesame model coefficients				Blackish Sesame model coefficients			
	WL (g) Model	Standard Error	t-value	P-value	WL (g) Model	Standard Error	t-value	P-value
Intercept	1.202069	0.110607	10.86793	0.000012	2.521034	0.056618	44.52711	0.000000
X_1 (L)	0.466667	0.108748	4.29127	0.003605	0.786667	0.055666	14.13182	0.000002
X_1 (Q)	-0.177241	0.160285	-1.10579	0.305367	-0.008621	0.082047	-0.10507	0.919268
X_2 (L)	0.416667	0.108748	3.83149	0.006445	0.320000	0.055666	5.74854	0.000699
X_2 (Q)	0.212759	0.160285	1.32738	0.226036	-0.088621	0.082047	-1.08012	0.315893
X_1 (L) × X_2 (Q)	0.062500	0.133188	0.46926	0.653149	0.060000	0.068177	0.88006	0.408022
Effect	Yellowish Sesame model coefficients				Blackish Sesame model coefficients			
	SK (g) Model	Standard Error	t-value	P-value	SK (g) Model	Standard Error	t-value	P-value
Intercept	66.05034	0.150878	437.7742	0.000000	69.19034	0.380569	181.8076	0.000000
X_1 (L)	1.42333	0.148342	9.5950	0.000028	-0.46667	0.374173	-1.2472	0.252434
X_1 (Q)	1.35379	0.218642	6.1918	0.000449	-0.76621	0.551497	-1.3893	0.207324
X_2 (L)	0.75833	0.148342	5.1121	0.001381	-0.36000	0.374173	-0.9621	0.368037
X_2 (Q)	-0.40121	0.218642	-1.8350	0.109144	0.30379	0.551497	0.5509	0.598867
X_1 (L) × X_2 (Q)	-0.50750	0.181681	-2.7934	0.026779	-0.23500	0.458266	-0.5128	0.623871

X_1: Heating temperature (°C), X_2: Heating time (min); L: Linear term; Q: Quadratic term; WL: Weight Loss; SK: Seedcake; P-value < 0.05 implies significant and P-value > 0.05 implies non-significant.

Table S8. Model parameter estimates (seedcake sediments in the oil, extraction loss, and extracted crude oil) and their statistical evaluation for sesame varieties.

Effect	Yellowish Sesame model coefficients				Blackish Sesame model coefficients			
	SD (g) Model	Standard Error	t-value	P-value	SD (g) Model	Standard Error	t-value	P-value
Intercept	5.647931	0.327195	17.26167	0.000001	5.423448	0.304923	17.78631	0.000000
X_1 (L)	0.226667	0.321696	0.70460	0.503820	1.056667	0.299797	3.52460	0.009668
X_1 (Q)	0.597241	0.474151	1.25960	0.248184	-0.702069	0.441875	-1.58884	0.156120
X_2 (L)	-0.353333	0.321696	-1.09835	0.308389	0.458333	0.299797	1.52881	0.170158
X_2 (Q)	0.537241	0.474151	1.13306	0.294501	-0.457069	0.441875	-1.03439	0.335363
X_1 (L) × X_2 (Q)	-0.277500	0.393995	-0.70432	0.503982	0.125000	0.367175	0.34044	0.743516
Effect	Yellowish Sesame model coefficients				Blackish Sesame model coefficients			
	EL (%) Model	Standard Error	t-value	P-value	EL (%) Model	Standard Error	t-value	P-value
Intercept	1.621876	0.215377	7.53041	0.000134	1.240405	0.143038	8.67189	0.000054

X_1 (L)	-0.295945	0.211757	-1.39757	0.204939	-0.163228	0.140633	-1.16066	0.283828
X_1 (Q)	-0.069123	0.312110	-0.22147	0.831048	0.257641	0.207281	1.24295	0.253902
X_2 (L)	-0.147814	0.211757	-0.69804	0.507671	-0.034215	0.140633	-0.24329	0.814754
X_2 (Q)	-0.719441	0.312110	-2.30509	0.054581	-0.162979	0.207281	-0.78627	0.457494
X_1 (L) × X_2 (Q)	0.323068	0.259348	1.24569	0.252952	-0.087020	0.172240	-0.50522	0.628917

Effect	Yellowish Sesame model coefficients				Blackish Sesame model coefficients			
	CO (g) Model	Standard Error	t-value	P-value	CO (g) Model	Standard Error	t-value	P-value
Intercept	25.49621	0.345639	73.76538	0.000000	21.65586	0.488417	44.33886	0.000000
X_1 (L)	-1.81833	0.339830	-5.35072	0.001063	-1.20667	0.480208	-2.51280	0.040232
X_1 (Q)	-1.70672	0.500879	-3.40746	0.011327	1.22448	0.707784	1.73002	0.127246
X_2 (L)	-0.67167	0.339830	-1.97648	0.088641	-0.38167	0.480208	-0.79479	0.452833
X_2 (Q)	0.36328	0.500879	0.72528	0.491810	0.39948	0.707784	0.56441	0.590092
X_1 (L) × X_2 (Q)	0.40250	0.416205	0.96707	0.365724	0.13500	0.588132	0.22954	0.825012

X_1: Heating temperature (°C), X_2: Heating time (min); L: Linear term; Q: Quadratic term; SD: Sediments; EL: Extraction Loss; CO: Extracted Crude Oil without seedcake sediments; P-value < 0.05 implies significant and P-value > 0.05 implies non-significant.

Table S9. Model parameter estimates (oil yield and oil expression efficiency) and their statistical evaluation for sesame varieties.

Effect	Yellowish Sesame model coefficients				Blackish Sesame model coefficients			
	OY (%) Model	Standard Error	t-value	P-value	OY (%) Model	Standard Error	t-value	P-value
Intercept	25.80603	0.345211	74.75438	0.000000	22.21522	0.494501	44.92454	0.000000
X_1 (L)	-1.72400	0.339409	-5.07942	0.001432	-1.04542	0.486189	-2.15023	0.068590
X_1 (Q)	-1.77801	0.500258	-3.55419	0.009291	1.24839	0.716600	1.74210	0.125025
X_2 (L)	-0.57439	0.339409	-1.69232	0.134421	-0.31402	0.486189	-0.64588	0.538948
X_2 (Q)	0.41875	0.500258	0.83707	0.430199	0.38540	0.716600	0.53781	0.607372
X_1 (L) × X_2 (Q)	0.41537	0.415689	0.99923	0.350967	0.15023	0.595458	0.25229	0.808063

Effect	Yellowish Sesame model coefficients				Blackish Sesame model coefficients			
	OEF (%) Model	Standard Error	t-value	P-value	OEF (%) Model	Standard Error	t-value	P-value
Intercept	66.63059	0.891327	74.75438	0.000000	49.02939	1.091372	44.92454	0.000000
X_1 (L)	-4.45133	0.876345	-5.07942	0.001432	-2.30726	1.073028	-2.15023	0.068590
X_1 (Q)	-4.59078	1.291655	-3.55419	0.009291	2.75522	1.581548	1.74210	0.125025
X_2 (L)	-1.48306	0.876345	-1.69232	0.134421	-0.69304	1.073028	-0.64588	0.538948
X_2 (Q)	1.08121	1.291655	0.83707	0.430199	0.85058	1.581548	0.53781	0.607372

X_1 (L) × X_2 (Q)	1.07247	1.073300	0.99923	0.350967	0.33156	1.314186	0.25229	0.808063

X_1: Heating temperature (°C), X_2: Heating time (min); L: Linear term; Q: Quadratic term; OY: Oil Yield and OEF: Oil Expression Efficiency; P-value < 0.05 implies significant and P-value > 0.05 implies non-significant.

Table S10. Analysis of variance for the weight loss and seedcake parameters (regression models) of sesame varieties.

	Yellowish Sesame: WL (g)					Blackish Sesame: WL (g)				
**Factor**	**SS**	**df**	**MS**	**F**	**p**	**SS**	**df**	**MS**	**F**	**p**
**Intercept**	2.519	5	0.504	7.099	0.011*	4.369	5	0.8739	47.002	0.000*
X_1 **(L)**	1.3067	1	1.3067	275.088	0.0001*	3.713	1	3.7131	270.632	0.000*
X_1 **(Q)**	0.0868	1	0.0868	18.266	0.0129*	0.0002	1	0.0002	0.0150	0.909**
X_2 **(L)**	1.0417	1	1.0417	219.298	0.0001*	0.614	1	0.6144	44.781	0.003*
X_2 **(Q)**	0.1250	1	0.1250	26.320	0.0068*	0.022	1	0.0217	1.581	0.277**
X_1 **(L) by** X_2 **(Q)**	0.0156	1	0.0156	3.289	0.1439**	0.014	1	0.0144	1.049	0.364**
**Residual**	0.497	7	0.071			0.130	7	0.019		
**Lack of Fit**	0.4777	3	0.1592	33.523	0.0027*	0.075	3	0.0251	1.829	0.282**
**Pure Error**	0.0190	4	0.0047			0.055	4	0.0137		
**Total SS**	3.0156	12				4.499	12			

	Yellowish Sesame: SK (g)					Blackish Sesame: SK (g)				
**Factor**	**SS**	**df**	**MS**	**F**	**p**	**SS**	**df**	**MS**	**F**	**p**
**Intercept**	21.740	5	4.348	32.932	0.000*	3.927	5	0.785	0.935	0.512*
X_1 **(L)**	12.155	1	12.155	76.386	0.001*	1.307	1	1.307	1.114	0.351**
X_1 **(Q)**	5.062	1	5.062	31.810	0.005*	1.621	1	1.621	1.382	0.305**
X_2 **(L)**	3.450	1	3.450	21.683	0.010*	0.778	1	0.778	0.663	0.461**
X_2 **(Q)**	0.445	1	0.445	2.794	0.170**	0.255	1	0.255	0.217	0.665**
X_1 **(L) by** X_2 **(Q)**	1.030	1	1.030	6.474	0.064*	0.221	1	0.221	0.188	0.687**

Residual	0.924	7	0.132			5.880	7	0.840		
**Lack of Fit**	0.288	3	0.096	0.603	0.647**	1.188	3	0.396	0.338	0.801**
**Pure Error**	0.637	4	0.159			4.693	4	1.173		
**Total SS**	22.664	12				9.808	12			

X_1: Heating temperature (°C), X_2: Heating time (min); L: Linear term; Q: Quadratic term; SS: Sum of Squares; df: Degrees of freedom; MS: Mean Squares; F-value: Compares variances and assesses the significance of model fits; P-value: Determines statistical significance; WL: Weight Loss; SK: Seedcake; SD: Sediments; * P-value < 0.05 or greater F-value means significant and ** P-value > 0.05 or lower F-value means non-significant.

Table S11. Analysis of variance for the seedcake sediments in the oil and percentage extraction loss parameters (regression models) of sesame varieties.

	Yellowish Sesame: SD (g)					Blackish Sesame: SD (g)				
**Factor**	**SS**	**df**	**MS**	**F**	**p**	**SS**	**df**	**MS**	**F**	**p**
**Intercept**	4.240	5	0.848	1.366	0.341**	11.079	5	2.2159	4.109	0.046*
X_1 (L)	0.308	1	0.308	1.775	0.254**	6.699	1	6.699	10.022	0.034*
X_1 (Q)	0.985	1	0.985	5.674	0.076**	1.361	1	1.361	2.037	0.227**
X_2 (L)	0.749	1	0.749	4.314	0.106**	1.260	1	1.260	1.886	0.242**
X_2 (Q)	0.797	1	0.797	4.591	0.099**	0.577	1	0.577	0.863	0.405**
X_1 (L) by X_2 (Q)	0.308	1	0.308	1.774	0.254**	0.063	1	0.063	0.094	0.775**
**Residual**	4.347	7	0.621			3.775	7	0.539		
**Lack of Fit**	3.652	3	1.217	7.011	0.045*	1.101	3	0.367	0.549	0.675**
**Pure Error**	0.695	4	0.174			2.674	4	0.669		
**Total SS**	8.587	12				14.854	12			
	**Yellowish Sesame: EL (%)**					**Blackish Sesame: EL (%)**				
**Factor**	**SS**	**df**	**MS**	**F**	**p**	**SS**	**df**	**MS**	**F**	**p**
**Intercept**	2.884	5	0.577	2.144	0.174**	0.394	5	0.079	0.664	0.663**
X_1 (L)	0.525	1	0.525	1.243	0.327**	0.159	1	0.159	0.838	0.412**
X_1 (Q)	0.013	1	0.013	0.031	0.868**	0.183	1	0.183	0.961	0.382**
X_2 (L)	0.131	1	0.131	0.310	0.607**	0.007	1	0.007	0.037	0.857**
X_2 (Q)	1.430	1	1.430	3.381	0.140**	0.073	1	0.073	0.385	0.569**
X_1 (L) by X_2 (Q)	0.417	1	0.417	0.987	0.377**	0.030	1	0.030	0.159	0.711**

**Residual**	1.883	7	0.269			0.831	7	0.119		
**Lack of Fit**	0.192	3	0.064	0.151	0.924**	0.068	3	0.023	0.118	0.945**
**Pure Error**	1.692	4	0.422			0.763	4	0.191		
**Total SS**	4.767	12				1.225	12			

X_1: Heating temperature (°C), X_2: Heating time (min); L: Linear term; Q: Quadratic term; SS: Sum of Squares; df: Degrees of freedom; MS: Mean Squares; F-value: Compares variances and assesses the significance of model fits; P-value: Determines statistical significance; SD: Sediments; EL: Extraction Loss; * P-value < 0.05 or greater F-value means significant and ** P-value > 0.05 or lower F-value means non-significant

Table S12. Analysis of variance for the extracted crude oil, oil yield, and oil expression efficiency parameters (regression models) of sesame varieties.

	**Yellowish Sesame: CO (g)**					**Blackish Sesame: CO (g)**				
**Factor**	**SS**	**df**	**MS**	**F**	**p**	**SS**	**df**	**MS**	**F**	**p**
**Intercept**	31.504	5	6.301	9.093	0.006*	16.247	5	3.249	2.349	0.148**
X_1 **(L)**	19.838	1	19.838	62.753	0.001*	8.736	1	8.736	5.628	0.077**
X_1 **(Q)**	8.045	1	8.045	25.449	0.007*	4.141	1	4.141	2.668	0.178**
X_2 **(L)**	2.707	1	2.707	8.562	0.043*	0.874	1	0.874	0.563	0.495**
X_2 **(Q)**	0.364	1	0.364	1.153	0.343**	0.441	1	0.441	0.284	0.622**
X_1 **(L) by** X_2 **(Q)**	0.648	1	0.648	2.050	0.225**	0.073	1	0.073	0.047	0.839**
**Residual**	4.850	7	0.693			9.685	7	1.384		
**Lack of Fit**	3.586	3	1.195	3.781	0.116**	3.476	3	1.159	0.746	0.578**
**Pure Error**	1.265	4	0.316			6.209	4	1.552		
**Total SS**	36.354	12				25.932	12			
	**Yellowish Sesame: OY (%)**					**Blackish Sesame: OY (%)**				
**Factor**	**SS**	**df**	**MS**	**F**	**p**	**SS**	**df**	**MS**	**F**	**p**
**Intercept**	29.450	5	5.890	8.522	0.007*	13.939	5	2.788	1.966	0.201**
X_1 **(L)**	17.833	1	17.833	52.224	0.002*	6.557	1	6.557	4.040	0.115**
X_1 **(Q)**	8.731	1	8.731	25.570	0.007*	4.304	1	4.304	2.652	0.179**
X_2 **(L)**	1.980	1	1.980	5.797	0.074**	0.592	1	0.592	0.365	0.579**
X_2 **(Q)**	0.484	1	0.484	1.418	0.300**	0.410	1	0.410	0.253	0.642**
X_1 **(L) by** X_2 **(Q)**	0.690	1	0.690	2.021	0.228**	0.090	1	0.090	0.056	0.825**

**Residual**	4.838	7	0.691			9.928	7	1.418		
**Lack of Fit**	3.472	3	1.157	3.390	0.135**	3.436	3	1.145	0.706	0.597**
**Pure Error**	1.366	4	0.341			6.492	4	1.6231		
**Total SS**	34.288	12				23.867	12			

	**Yellowish Sesame: OEF (%)**					**Blackish Sesame: OEF (%)**				
**Factor**	**SS**	**df**	**MS**	**F**	**p**	**SS**	**df**	**MS**	**F**	**p**
**Intercept**	196.332	5	39.266	8.522	0.007	67.894	5	13.579	1.966	0.201**
**X_1 (L)**	118.886	1	118.886	52.224	0.002*	31.941	1	31.941	4.040	0.115**
**X_1 (Q)**	58.208	1	58.208	25.570	0.007*	20.966	1	20.966	2.652	0.179**
**X_2 (L)**	13.197	1	13.197	5.797	0.074**	2.882	1	2.882	0.365	0.579**
**X_2 (Q)**	3.229	1	3.229	1.418	0.300**	1.9982	1	1.998	0.253	0.642**
**X_1 (L) by X_2 (Q)**	4.601	1	4.601	2.021	0.228**	0.439	1	0.439	0.056	0.825**
**Residual**	32.255	7	4.608			48.358	7	6.908		
**Lack of Fit**	23.149	3	7.716	3.390	0.135**	16.735	3	5.578	0.706	0.597**
**Pure Error**	9.106	4	2.276			31.624	4	7.906		
**Total SS**	228.587	12				116.252	12			

X_1: Heating temperature (°C), X_2: Heating time (min); L: Linear term; Q: Quadratic term; SS: Sum of Squares; df: Degrees of freedom; MS: Mean Squares; F-value: Compares variances and assesses the significance of model fits; P-value: Determines statistical significance; CO: Extracted Crude Oil without seedcake sediments; OY: Oil Yield; OEF: Oil Expression Efficiency; * P-value < 0.05 or greater F-value means significant and ** P-value > 0.05 or lower F-value means non-significant.

Table S13. Observed, predicted, and residual results of the determined parameters of yellowish sesame based on the regression coefficients (Eq. 4, Table 10).

Run	WL (g)			SK (g)			SD (g)			EL (%)		
	Obs.	Pred.	Resid.	Obs.	Pred.	Resid.	Obs.	Pred.	Resid.	Obs.	Pred.	Resid.
1	0.45	0.42	0.03	64.2	64.31	-0.11	6.95	6.63	0.32	1.46	1.60	-0.14
2	0.72	0.56	0.16	66.13	65.98	0.15	6.31	6.02	0.29	1.90	1.85	0.06
3	0.93	1.13	-0.20	66.81	66.85	-0.04	5.87	6.48	-0.61	0.75	0.66	0.09
4	1.00	1.00	0.00	64.86	64.89	-0.03	5.39	6.54	-1.15	1.35	1.05	0.30
5	1.19	1.20	-0.01	65.73	66.05	-0.32	5.49	5.65	-0.16	1.21	1.62	-0.41
6	2.29	1.83	0.46	66.22	66.41	-0.19	6.54	5.83	0.71	0.59	0.75	-0.16
7	1.19	1.23	-0.04	68.32	68.18	0.14	8.47	7.64	0.83	0.20	0.36	-0.16
8	1.79	1.49	0.30	68.46	68.83	-0.37	5.74	6.47	-0.73	1.34	1.26	0.09
9	1.92	2.18	-0.26	68.9	68.68	0.22	6.28	6.38	-0.10	0.79	0.71	0.07
10	1.13	1.20	-0.07	66.74	66.05	0.69	5.97	5.65	0.32	0.84	1.62	-0.78
11	1.00	1.20	-0.20	65.8	66.05	-0.25	5.9	5.65	0.25	2.42	1.62	0.80

12	1.12	1.20	-0.08	66.09	66.05	0.04	5.14	5.65	-0.51	2.09	1.62	0.47
13	1.11	1.20	-0.09	66.11	66.05	0.06	6.18	5.65	0.53	1.40	1.62	-0.23

	CO (g)			OY (%)			OEF (%)		
Run	Obs.	Pred.	Resid.	Obs.	Pred.	Resid.	Obs.	Pred.	Resid.
1	26.95	27.05	-0.10	27.07	27.16	-0.09	69.90	70.13	-0.23
2	24.95	25.61	-0.66	25.13	25.75	-0.62	64.89	66.49	-1.60
3	25.65	24.90	0.75	25.89	25.18	0.71	66.85	65.02	1.83
4	27.41	26.53	0.88	27.69	26.80	0.89	71.49	69.19	2.29
5	26.39	25.50	0.89	26.71	25.81	0.90	68.96	66.63	2.33
6	24.37	25.19	-0.82	24.94	25.65	-0.71	64.40	66.23	-1.83
7	21.82	22.60	-0.78	22.08	22.88	-0.80	57.02	59.08	-2.06
8	22.69	21.97	0.72	23.10	22.30	0.80	59.65	57.59	2.06
9	22.13	22.07	0.06	22.56	22.56	0.00	58.26	58.26	0.00
10	25.33	25.50	-0.17	25.62	25.81	-0.19	66.15	66.63	-0.48
11	24.9	25.50	-0.60	25.15	25.81	-0.65	64.94	66.63	-1.69
12	25.58	25.50	0.08	25.87	25.81	0.06	66.80	66.63	0.16
13	25.22	25.50	-0.28	25.50	25.81	-0.30	65.85	66.63	-0.78

WL: Weight Loss; SK: Seedcake; SD: Sediments; EL: Extraction Loss; CO: Extracted Crude Oil without seedcake sediments; OY: Oil Yield; OEF: Oil Expression Efficiency; Obs.: Observed data; Pred.: Predicted data, and Resids.: Residuals.

Table S14. Observed, predicted, and residual results of the determined parameters of blackish sesame varieties based on the regression coefficients (Eq. 4, Table 10).

	WL (g)			SK (g)			SD (g)			EL (%)		
Run	Obs.	Pred.	Resid.	Obs.	Pred.	Resid.	Obs.	Pred.	Resid.	Obs.	Pred.	Resid.
1	1.48	1.38	0.10	69.21	69.32	-0.11	3.33	2.87	0.46	1.52	1.45	0.08
2	1.66	1.73	-0.07	69.35	68.89	0.46	3.11	3.66	-0.55	1.49	1.66	-0.17
3	1.86	1.90	-0.04	68.72	69.07	-0.35	3.64	3.54	0.10	1.64	1.55	0.09
4	1.92	2.11	-0.19	69.51	69.85	-0.34	3.85	4.51	-0.66	1.03	1.11	-0.08
5	2.6	2.52	0.08	69.37	69.19	0.18	4.09	5.42	-1.33	1.16	1.24	-0.08
6	2.84	2.75	0.09	69.27	69.13	0.14	5.48	5.42	0.06	0.94	1.04	-0.11
7	2.92	2.83	0.09	69.31	68.86	0.45	4.94	4.74	0.20	1.30	1.29	0.00
8	3.26	3.30	-0.04	67.29	67.96	-0.67	5.73	5.78	-0.05	1.31	1.33	-0.02
9	3.54	3.59	-0.05	67.88	67.67	0.21	5.75	5.90	-0.15	1.07	1.05	0.02
10	2.49	2.52	-0.03	70.39	69.19	1.20	5.77	5.42	0.35	1.64	1.24	0.40
11	2.64	2.52	0.12	69.77	69.19	0.58	5.99	5.42	0.57	0.77	1.24	-0.47
12	2.62	2.52	0.10	69.14	69.19	-0.05	5.98	5.42	0.56	1.01	1.24	-0.23
13	2.36	2.52	-0.16	67.49	69.19	-1.70	5.89	5.42	0.47	1.81	1.24	0.57

	CO (g)			OY (%)			OEF (%)		

Run	Obs.	Pred.	Resid.	Obs.	Pred.	Resid.	Obs.	Pred.	Resid.
1	24.48	25.00	-0.52	24.85	25.36	-0.51	54.84	55.97	-1.13
2	24.41	24.09	0.32	24.82	24.51	0.31	54.78	54.09	0.69
3	24.17	23.97	0.20	24.63	24.43	0.20	54.35	53.92	0.44
4	23.71	22.44	1.27	24.17	22.91	1.26	53.35	50.57	2.78
5	22.81	21.66	1.15	23.42	22.22	1.20	51.69	49.03	2.66
6	21.5	21.67	-0.17	22.13	22.29	-0.16	48.84	49.19	-0.35
7	21.57	22.32	-0.75	22.22	22.97	-0.75	49.04	50.69	-1.65
8	22.45	21.67	0.78	23.21	22.42	0.79	51.22	49.48	1.74
9	21.8	21.83	-0.03	22.60	22.64	-0.04	49.88	49.97	-0.09
10	19.75	21.66	-1.91	20.25	22.22	-1.96	44.70	49.03	-4.33
11	20.85	21.66	-0.81	21.42	22.22	-0.80	47.26	49.03	-1.77
12	21.28	21.66	-0.38	21.85	22.22	-0.36	48.23	49.03	-0.80
13	22.49	21.66	0.83	23.03	22.22	0.82	50.84	49.03	1.81

$\boldsymbol{WL}$: Weight Loss; $\boldsymbol{SK}$: Seedcake; $\boldsymbol{SD}$: Sediments; $\boldsymbol{EL}$: Extraction Loss; $\boldsymbol{CO}$: Extracted Crude Oil without seedcake sediments; $\boldsymbol{OY}$: Oil Yield; $\boldsymbol{OEF}$: Oil Expression Efficiency; Obs.: Observed data; Pred.: Predicted data, and Resids.: Residuals.

Table S15. Shapiro-Wilk test of normality of residuals of the parameters of yellowish and blackish sesame varieties based on the regression coefficients (Eq. 4, Table 10).

Yellowish sesame parameters							
Shapiro-Wilk test	$\boldsymbol{WL}$ (g)	$\boldsymbol{SK}$ (g)	$\boldsymbol{SD}$ (g)	$\boldsymbol{EL}$ (%)	$\boldsymbol{CO}$ (g)	$\boldsymbol{OY}$ (%)	$\boldsymbol{OEF}$ (%)
P-value	0.1977	0.2692	0.5636	0.8768	0.1247	0.0863	0.0863
R^2	0.9124	0.9223	0.9477	0.9686	0.8978	0.8861	0.8861
Blackish sesame parameters							
Shapiro-Wilk test	$\boldsymbol{WL}$ (g)	$\boldsymbol{SK}$ (g)	$\boldsymbol{SD}$ (g)	$\boldsymbol{EL}$ (%)	$\boldsymbol{CO}$ (g)	$\boldsymbol{OY}$ (%)	$\boldsymbol{OEF}$ (%)
P-value	0.0716	0.4249	0.0736	0.3238	0.8175	0.7329	0.7329
R^2	0.8801	0.9375	0.881	0.9283	0.9642	0.9587	0.9587

$\boldsymbol{WL}$: Weight Loss; $\boldsymbol{SK}$: Seedcake; $\boldsymbol{SD}$: Sediments; $\boldsymbol{EL}$: Extraction Loss; $\boldsymbol{CO}$: Extracted Crude Oil without seedcake sediments; $\boldsymbol{OY}$: Oil Yield; $\boldsymbol{OEF}$: Oil Expression Efficiency; P-value > 0.05 means data follow normality function and R^2: Coefficient of determination.

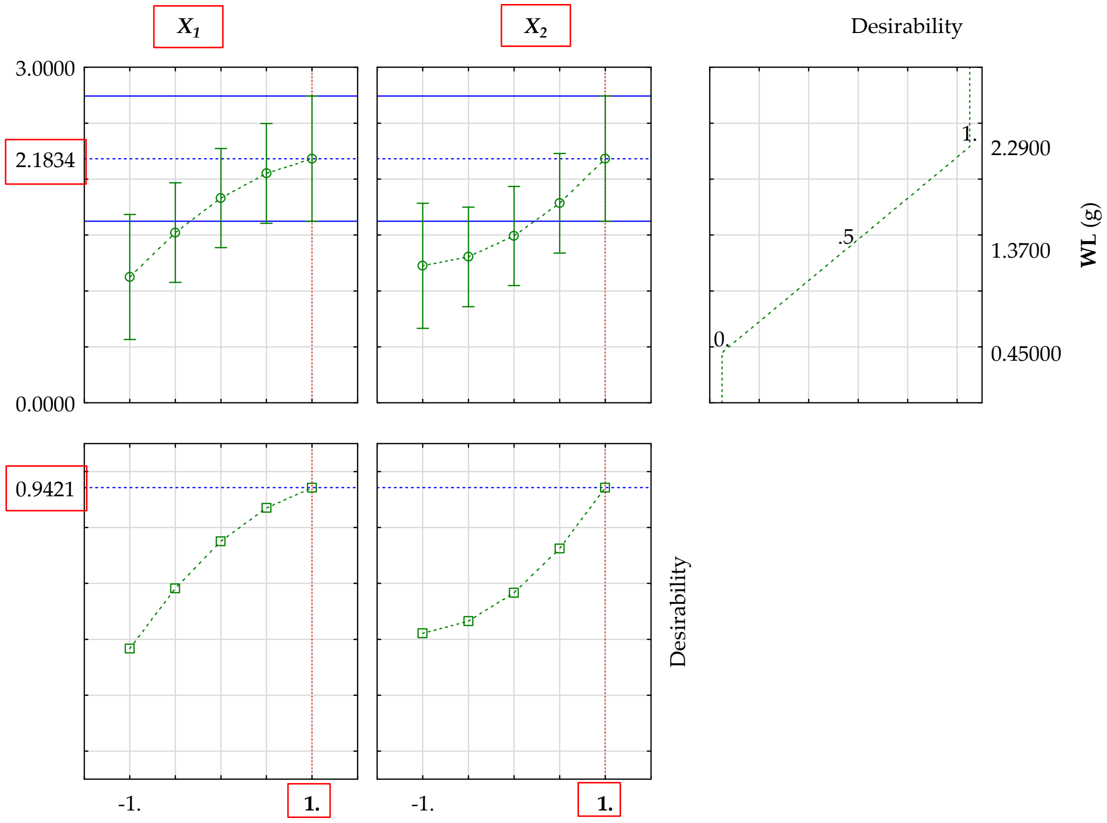

Figure S1. Profiles for predicted values and desirability of the factors' effect on weight loss (**WL**) of yellowish sesame ($\mathbf{X_1}$: heating temperature; $\mathbf{X_2}$: heating time; the blue grid lines indicate the optimal and desirability values of the dependent parameter, and the red gridlines indicate the factor levels; coded values **+1**: 60 °C and **+1**: 60 min).

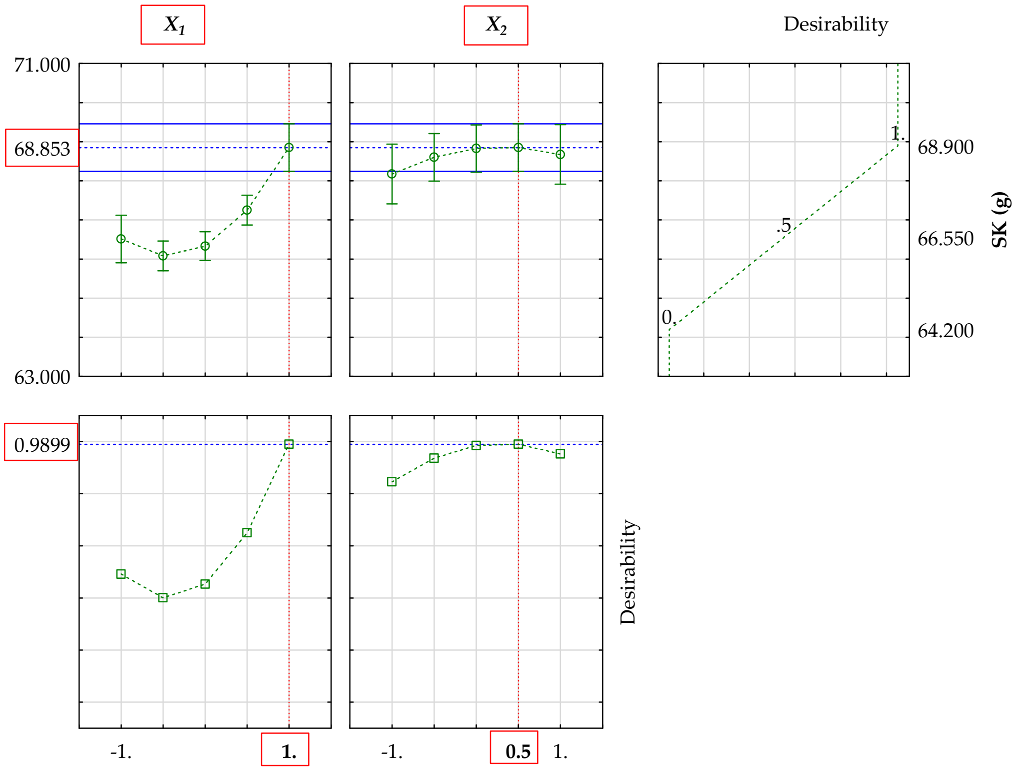

Figure S2. Profiles for predicted values and desirability of the factors' effect on seedcake (**SK**) of yellowish sesame ($\boldsymbol{X_1}$: heating temperature; $\boldsymbol{X_2}$: heating time; the blue grid lines indicate the optimal and desirability values of the dependent parameter, and the red gridlines indicate the factor levels; coded values +1: 60 °C and +0.5: 37.5 min).

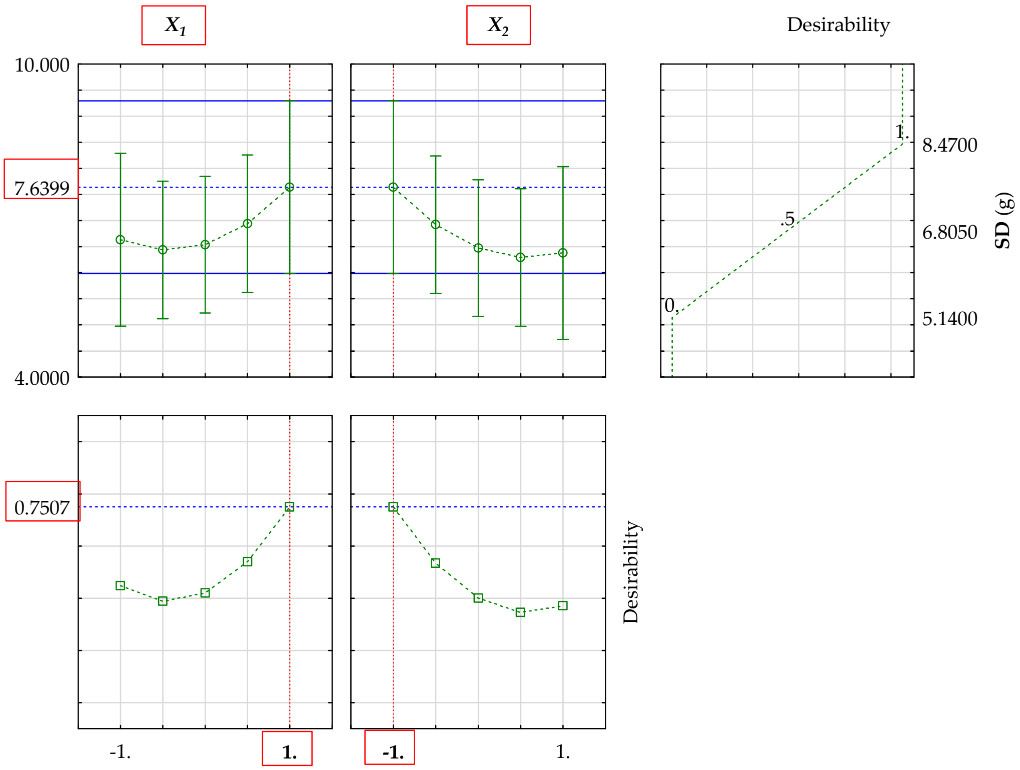

Figure S3. Profiles for predicted values and desirability of the factors' effect on seedcake sediments (***SD***) of yellowish sesame ($\boldsymbol{X_1}$: heating temperature; $\boldsymbol{X_2}$: heating time; the blue grid lines indicate the optimal and desirability values of the dependent parameter, and the red gridlines indicate the factor levels; coded values **+1**: 60 °C and **–1**: 15 min).

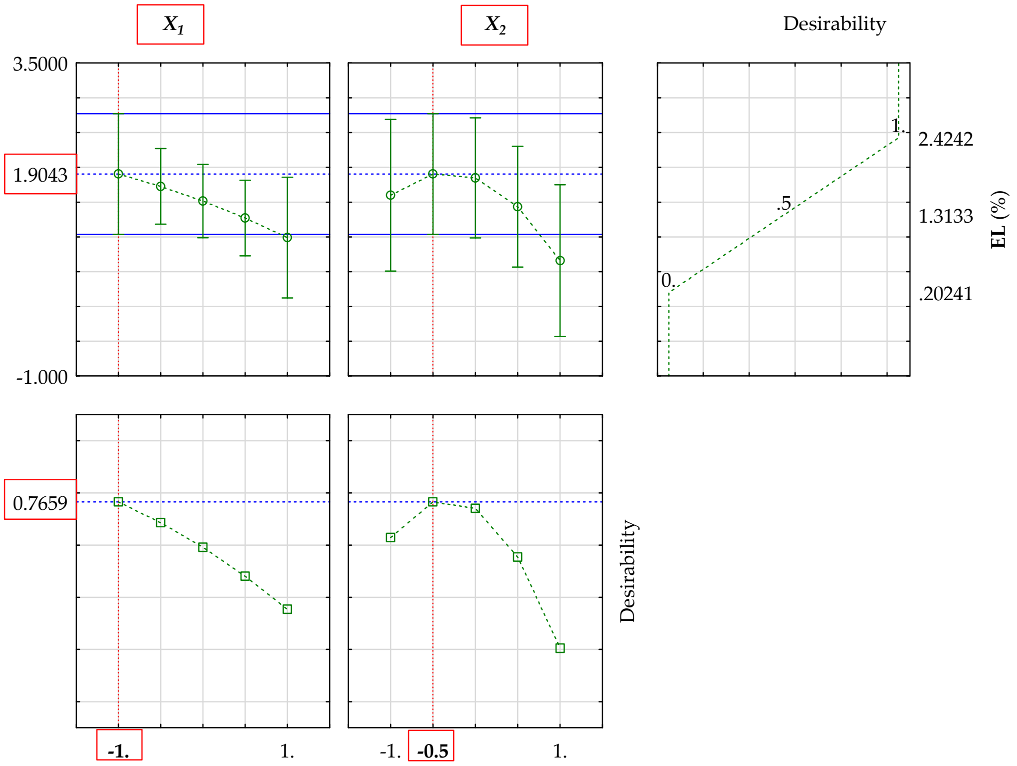

Figure S4. Profiles for predicted values and desirability of the factors' effect on extraction loss ($\boldsymbol{EL}$) of yellowish sesame ($\boldsymbol{X_1}$: heating temperature; $\boldsymbol{X_2}$: heating time; the blue grid lines indicate the optimal and desirability values of the dependent parameter, and the red gridlines indicate the factor levels; coded values **–1**: 40 °C and **–0.5**: 22.5 min).

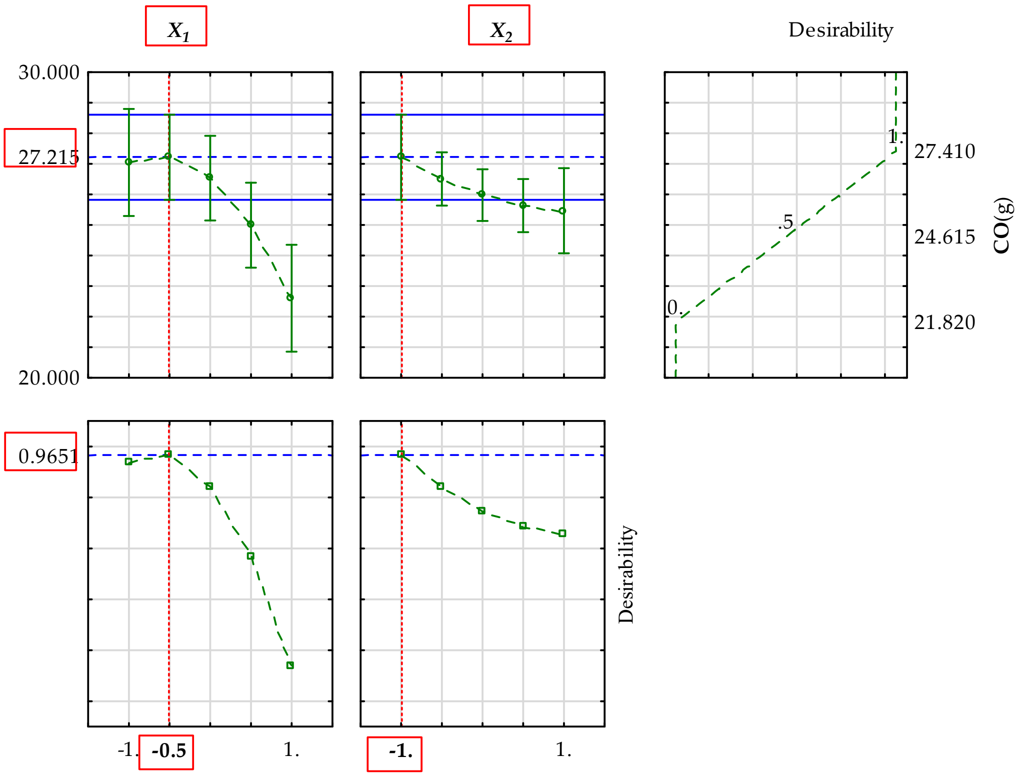

Figure S5. Profiles for predicted values and desirability of the factors' effect on extracted crude oil ($\boldsymbol{CO}$) of yellowish sesame ($\boldsymbol{X_1}$: heating temperature; $\boldsymbol{X_2}$: heating time; the blue grid lines indicate the optimal and desirability values of the dependent parameter, and the red gridlines indicate the factor levels; coded values **–0.5**: 45 °C and **–1**: 15 min).

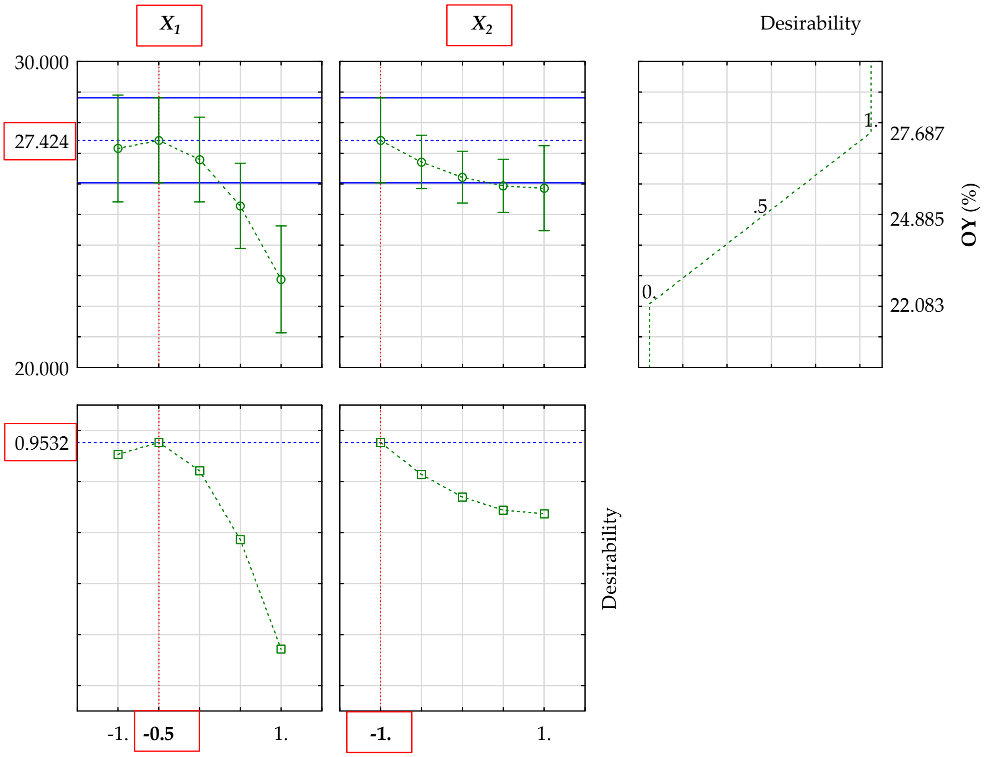

Figure S6. Profiles for predicted values and desirability of the factors' effect on oil yield (OY) of yellowish sesame (X_1: heating temperature; X_2: heating time; the blue grid lines indicate the optimal and desirability values of the dependent parameter and the red gridlines indicate the factor levels; coded values **–0.5**: 45 °C and **–1**: 15 min).

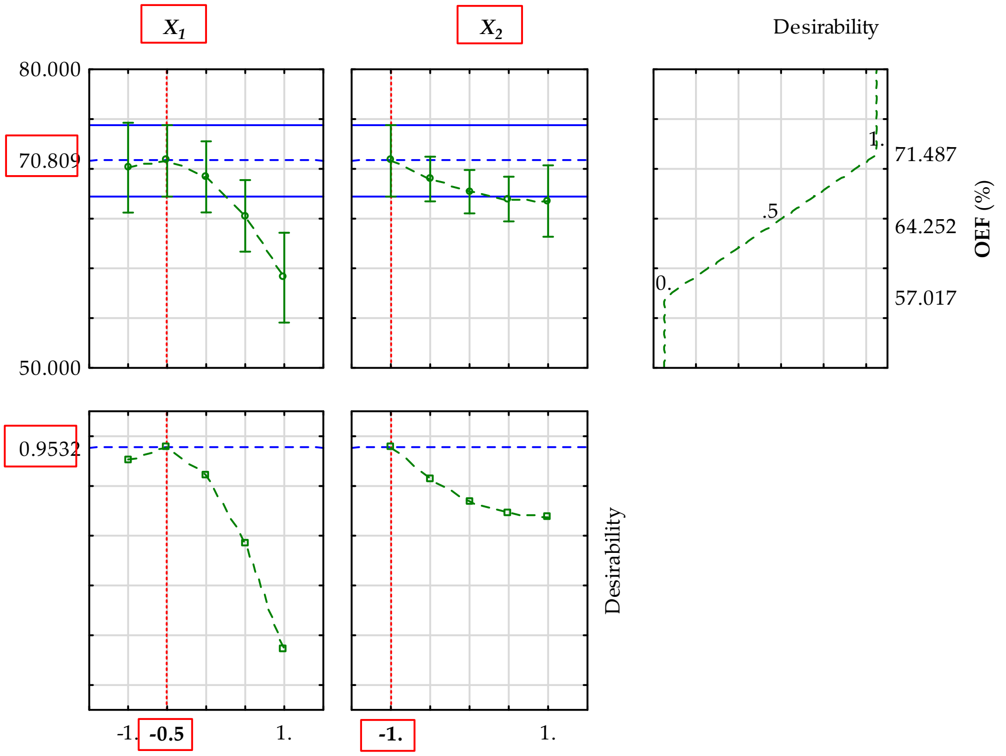

Figure S7. Profiles for predicted values and desirability of the factors' effect on oil expression efficiency (OEF) of yellowish sesame (X_1: heating temperature; X_2: heating time; the blue grid lines indicate the optimal and desirability values of the dependent parameter and the red gridlines indicate the factor levels; coded values **–0.5**: 45 °C and **–1**: 15 min).

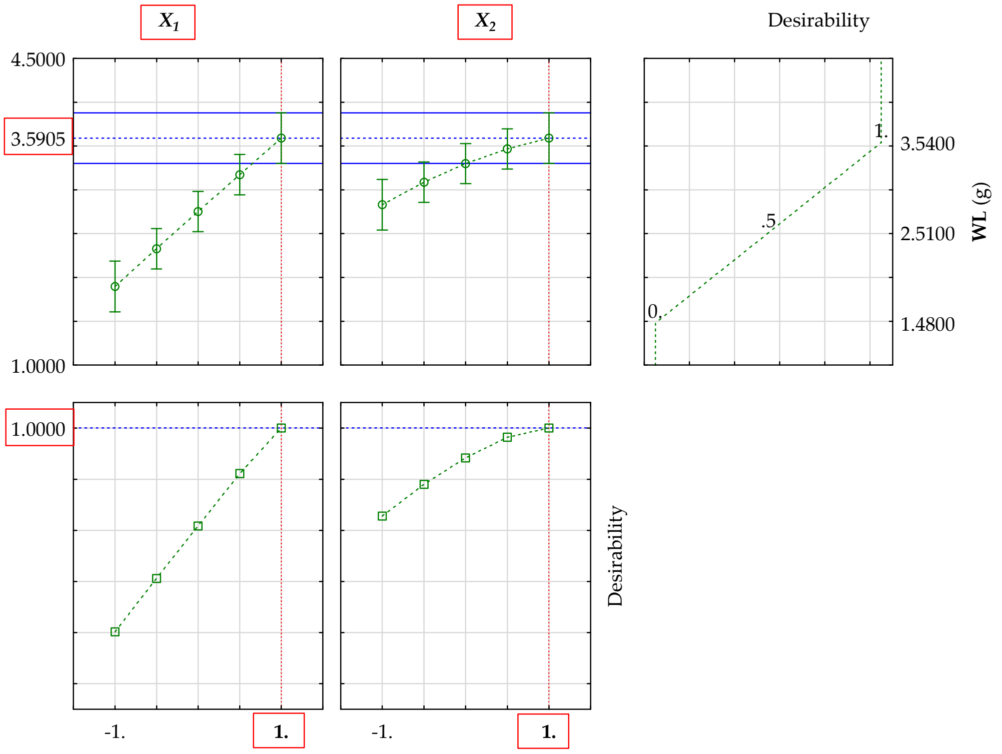

Figure S8. Profiles for predicted values and desirability of the factors' effect on weight loss ($\boldsymbol{WL}$) of blackish sesame ($\boldsymbol{X_1}$: heating temperature; $\boldsymbol{X_2}$: heating time; the blue grid lines indicate the optimal and desirability values of the dependent parameter, and the red gridlines indicate the factor levels; coded values **+1**: 60 °C and **+1**: 45 min).

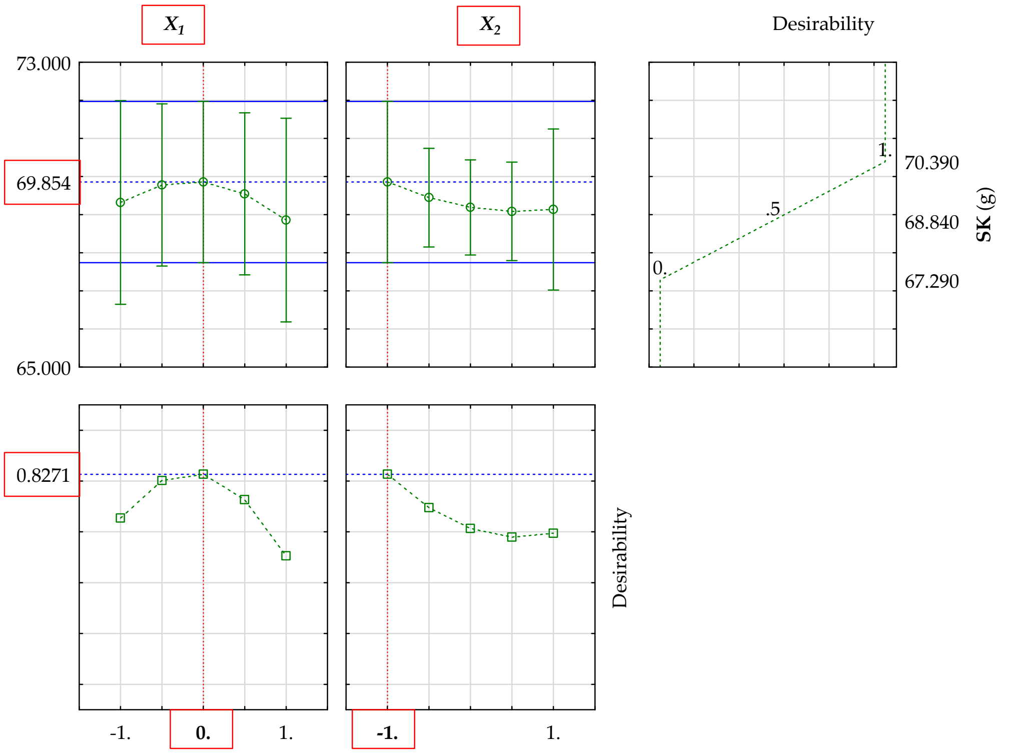

Figure S9. Profiles for predicted values and desirability of the factors' effect on seedcake ($\boldsymbol{SK}$) of blackish sesame ($\boldsymbol{X_1}$: heating temperature; $\boldsymbol{X_2}$: heating time; the blue grid lines indicate the optimal and desirability values of the dependent parameter, and the red gridlines indicate the factor levels; coded values **0**: 50 °C and **–1**: 15 min).

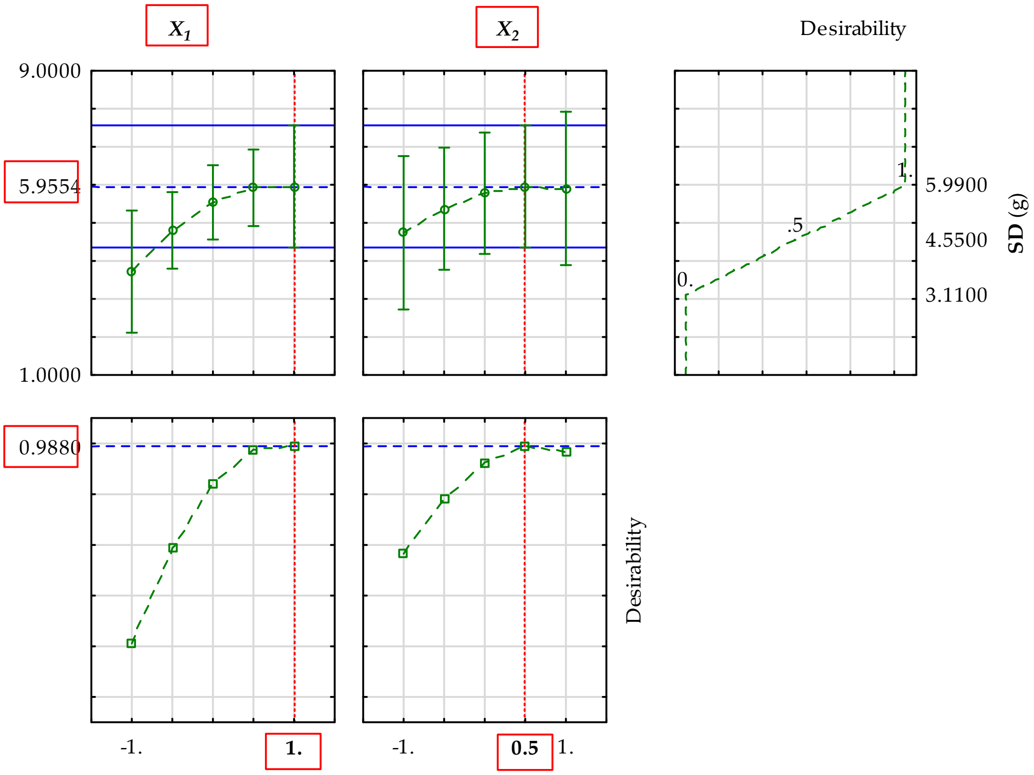

Figure S10. Profiles for predicted values and desirability of the factors' effect on seedcake sediments (***SD***) of blackish sesame ($\boldsymbol{X_1}$: heating temperature; $\boldsymbol{X_2}$: heating time; the blue grid lines indicate the optimal and desirability values of the dependent parameter, and the red gridlines indicate the factor levels; coded values **+1**: 60 °C and **0.5**: 37.5 min).

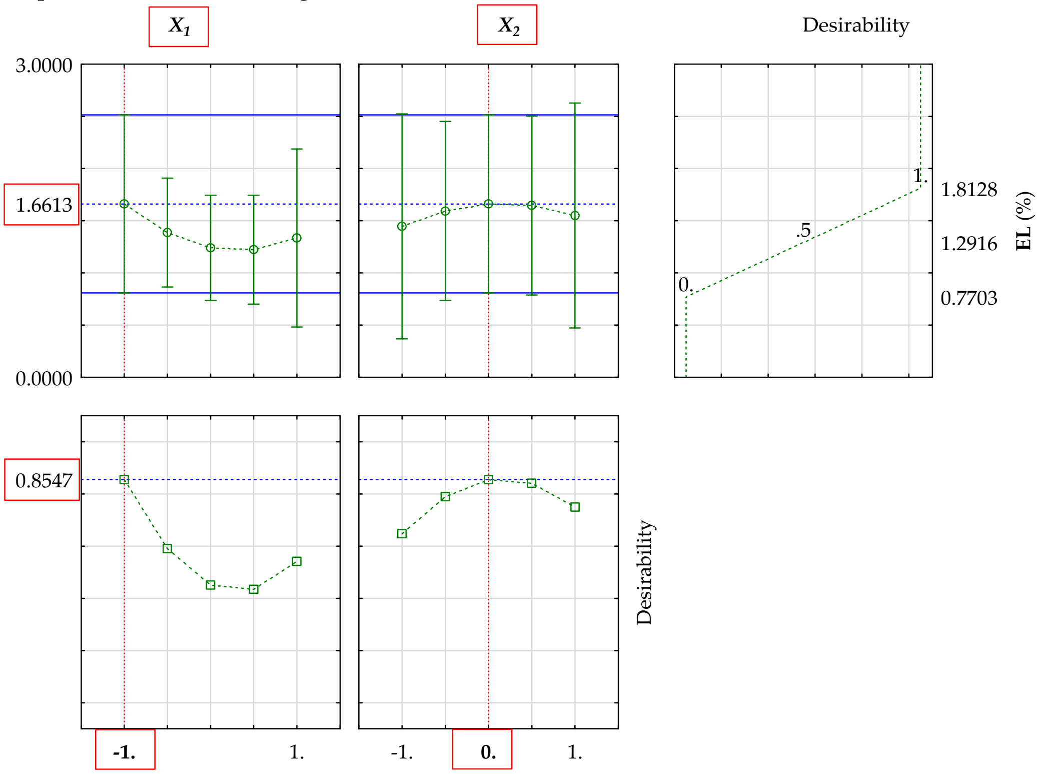

Figure S11. Profiles for predicted values and desirability of the factors' effect on extraction loss (***EL***) of blackish sesame ($\boldsymbol{X_1}$: heating temperature; $\boldsymbol{X_2}$: heating time; the blue grid lines indicate the optimal and desirability values of the dependent parameter, and the red gridlines indicate the factor levels; coded values **–1**: 40 °C and **0**: 30 min).

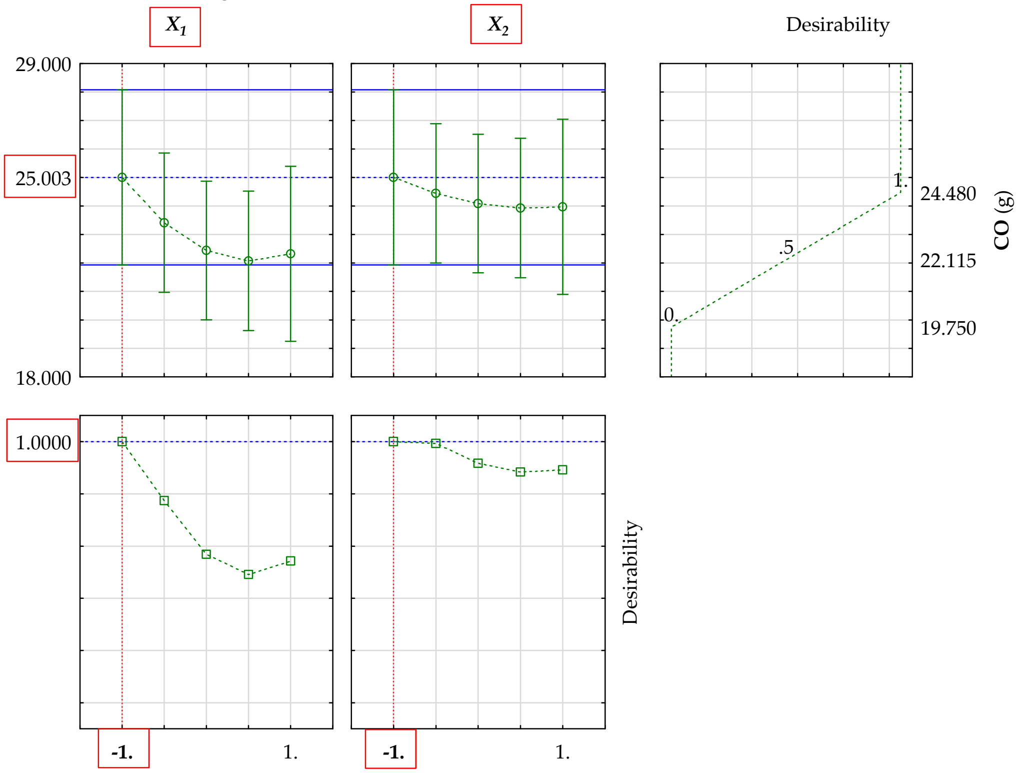

Figure S12. Profiles for predicted values and desirability of the factors' effect on extracted crude oil ($\boldsymbol{CO}$) of blackish sesame ($\boldsymbol{X_1}$: heating temperature; $\boldsymbol{X_2}$: heating time; the blue grid lines indicate the optimal and desirability values of the dependent parameter, and the red gridlines indicate the factor levels; coded values **–1**: 40 °C and **–1**: 15 min).

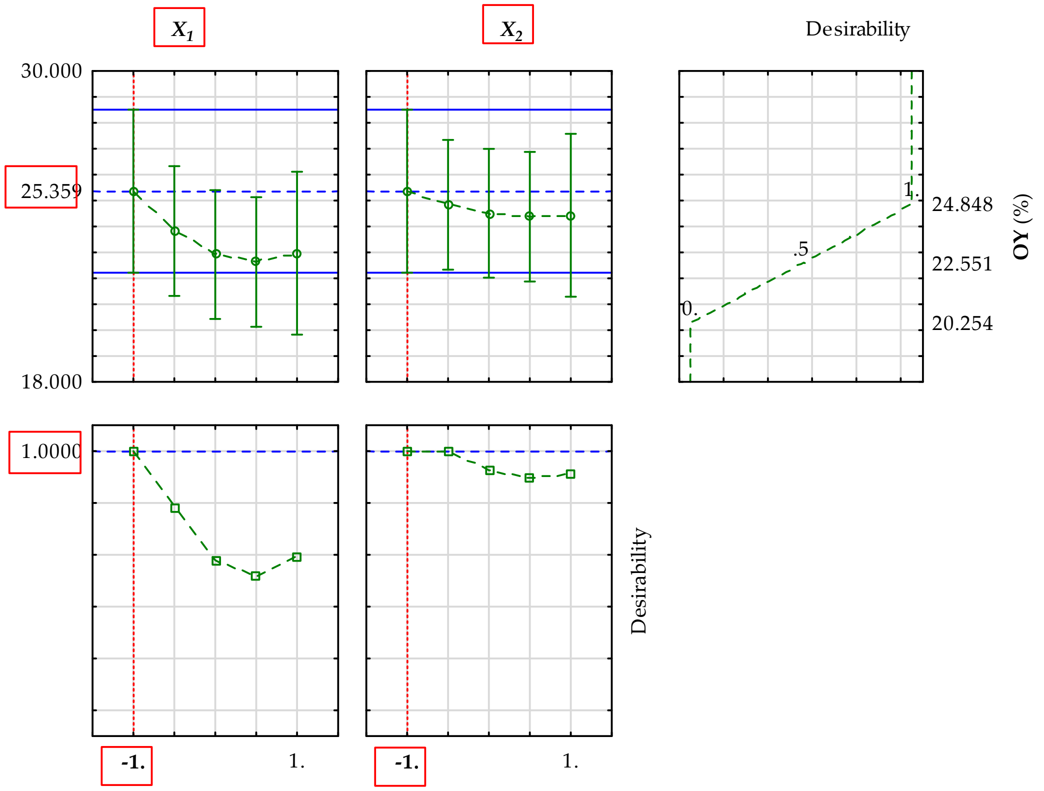

Figure S13. Profiles for predicted values and desirability of the factors' effect on oil yield ($\boldsymbol{OY}$) of blackish sesame ($\boldsymbol{X_1}$: heating temperature; $\boldsymbol{X_2}$: heating time; the blue grid lines indicate the optimal and desirability values of the dependent parameter, and the red gridlines indicate the factor levels; coded values **–1**: 40 °C and **–1**: 15 min).

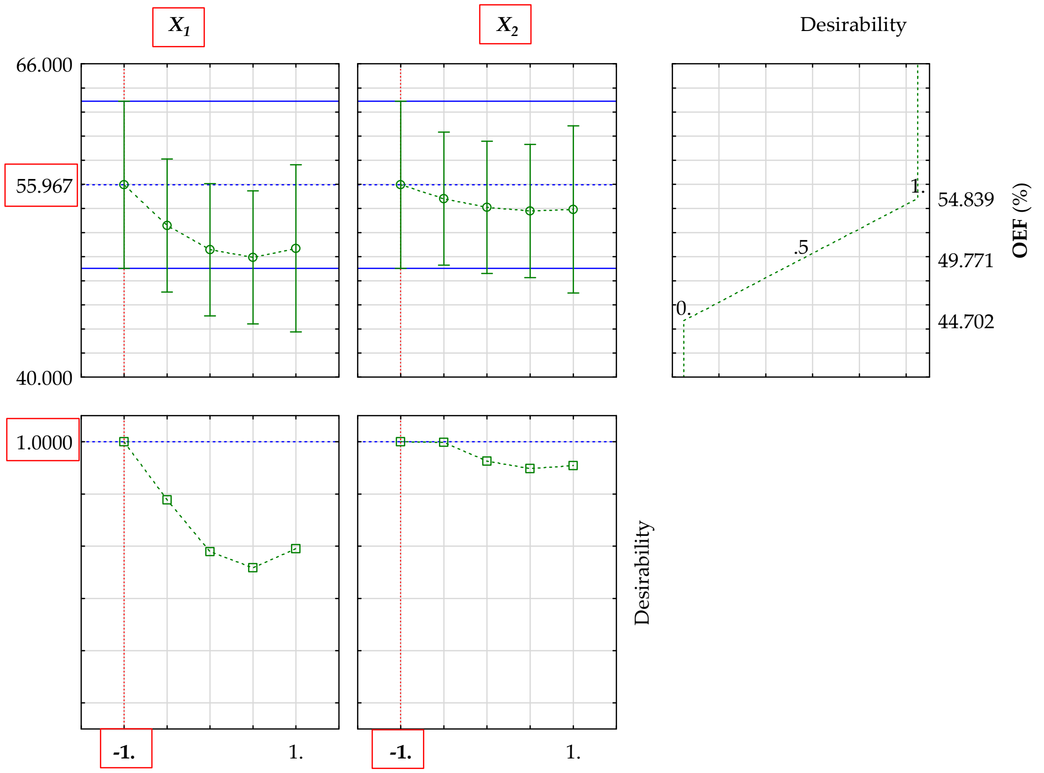

Figure S14 Profiles for predicted values and desirability of the factors' effect on oil expression efficiency ($\boldsymbol{OEF}$) of blackish sesame ($\boldsymbol{X_1}$: heating temperature; $\boldsymbol{X_2}$: heating time; the blue grid lines indicate the optimal and desirability values of the dependent parameter, and the red gridlines indicate the factor levels; coded values **–1**: 40 °C and **–1**: 15 min).

Disclaimer/Publisher's Note: The statements, opinions and data contained in all publications are solely those of the individual author(s) and contributor(s) and not of MDPI and/or the editor(s). MDPI and/or the editor(s) disclaim responsibility for any injury to people or property resulting from any ideas, methods, instructions or products referred to in the content.
